# Rock-Hosted Subsurface Biofilms: Mineral Selectivity Drives Hotspots for Intraterrestrial Life

**DOI:** 10.3389/fmicb.2021.658988

**Published:** 2021-04-09

**Authors:** Caitlin P. Casar, Brittany R. Kruger, Magdalena R. Osburn

**Affiliations:** ^1^Department of Earth and Planetary Sciences, Northwestern University, Evanston, IL, United States; ^2^Division of Hydrologic Sciences, Desert Research Institute, Las Vegas, NV, United States

**Keywords:** biofilm, mineral selectivity, deep subsurface, continental subsurface, biomass

## Abstract

The continental deep subsurface is likely the largest reservoir of biofilm-based microbial biomass on Earth, but the role of mineral selectivity in regulating its distribution and diversity is unclear. Minerals can produce hotspots for intraterrestrial life by locally enhancing biofilm biomass. Metabolic transformations of minerals by subsurface biofilms may occur widely with the potential to significantly impact subsurface biogeochemical cycles. However, the degree of impact depends upon the amount of biofilm biomass and its relationship to host rock mineralogy, estimates that are currently loosely constrained to non-existent. Here, we use *in situ* cultivation of biofilms on native rocks and coupled microscopy/spectroscopy to constrain mineral selectivity by biofilms in a deep continental subsurface setting: the Deep Mine Microbial Observatory (DeMMO). Through hotspot analysis and spatial modeling approaches we find that mineral distributions, particularly those putatively metabolized by microbes, indeed drive biofilm distribution at DeMMO, and that bioleaching of pyrite may be a volumetrically important process influencing fluid geochemistry at this site when considered at the kilometer scale. Given the ubiquity of iron-bearing minerals at this site and globally, and the amount of biomass they can support, we posit that rock-hosted biofilms likely contribute significantly to subsurface biogeochemical cycles. As more data becomes available, future efforts to estimate biomass in the continental subsurface should incorporate host rock mineralogy.

## Introduction

Continental deep subsurface ecosystems are spatially heterogeneous from the kilometer to micron scale. Here, the juxtaposition of different rock types and lithologies produces a buffet of mineral types and geochemical disequilibria that can support microbial metabolisms. Groundwaters flowing through aquifers connect diverse rock types, and water-rock interactions produce distinctive fracture fluid chemistry reflecting that of the surrounding rock, e.g. (Osburn et al., 2019). These fluids contain sources of energy and nutrients for microbial life and act as vectors for microbial transport throughout the subsurface ecosystem. This fluid movement is important for the lifecycle of biofilm-forming members of the biosphere, where dispersed cells can colonize optimal rock surfaces for attachment (Watnick and Kolter, 2000).

Mineral composition of rocks likely drives selective attachment by mineral-metabolizing microbes (Casar et al., 2020). Microbes exchange electrons with extracellular minerals in a number of ways including conductive appendages and chelating compounds, which can result in mineral dissolution (Shi et al., 2016). The active dissolution of minerals by microbial communities can enhance the mobilization of elements, thus altering local fluid chemistry (Ehrlich, 1996; Yan et al., 2019); however, the specific mechanisms for and products of these mineral transformations depend upon the local geochemistry and metabolic interplay between the attached and planktonic communities (Kwon et al., 2014). Given that continental subsurface biofilms likely comprise as much as 20-80% of total subsurface biomass (as many as 2.4 × 10^29^ cells) (Magnabosco et al., 2018; Flemming and Wuertz, 2019), these mineral transformations may occur on large scales and critically affect subsurface biogeochemical cycles.

Estimating the contribution of biofilm communities to subsurface biogeochemical cycles hinges on constraining estimates of biofilm biomass. Theoretical and experimental data suggest that a significant amount of biofilm biomass can be supported by growth on iron or manganese-bearing minerals in deep continental settings (Casar et al., 2020). However, these minerals are heterogeneously distributed through host rocks and therefore biofilm distribution is likely also heterogeneous, producing hotspots of microbial life selectively colonizing energy-rich minerals. Although mineral selectivity may be an important consideration for estimating biofilm biomass, current models estimating deep continental biomass do not take host rock mineralogy into account, owing in part to lack of existing data (Magnabosco et al., 2018; Flemming and Wuertz, 2019). Probing mineral selectivity in deep continental settings can provide a foundation for future incorporation of mineralogy into refined biomass estimates.

Mineral selectivity by biofilms is well-documented in surficial settings such as soil ecosystems (Ahmed and Holmström, 2015), but has not been thoroughly explored in the continental deep subsurface. Despite their ubiquity, subsurface biofilms are challenging to observe *in situ*. Biofilms can be physically detached via chiseling or drilling of rocks; however, this is a destructive and challenging sampling method. Biofilms can be cultivated in the laboratory using growth experiments with minerals inoculated with environmental fluids; however, this is also challenging due to the difficulty of mimicking the natural environment. Alternatively, biofilms can be grown *in situ* on experimental substrates seeded by environmental fluids that can be collected without significant alteration of the environment. Such *in situ* growth experiments have provided key insights into the metabolic relationship between biofilms and their attachment surfaces by allowing for controlled introduction of individual minerals or rock as attachment surfaces while maintaining otherwise native conditions (Moser et al., 2003; Lehman et al., 2004; Casar et al., 2020; Mullin et al., 2020; Rowe et al., 2020).

*In situ* subsurface growth experiments offer a way to constrain biofilm mineral selectivity. Previous experiments comparing single crystal energy-rich vs. energy-poor minerals suggest mineral selectivity is likely a key process regulating biofilm distribution and biomass in the continental deep subsurface (Casar et al., 2020). This previous work, carried out at the Deep Mine Microbial Observatory (DeMMO), defined energy-rich minerals as those in metabolic reactions that have the potential to be energy-yielding under *in situ* conditions, including pyrite, pyrolusite, and siderite. These minerals supported higher cell densities compared to energy-poor minerals such as gypsum, hematite, and magnetite, as well as inert glass controls. Further, biofilms growing on energy-rich minerals were enriched in taxa capable of utilizing solid growth substrates, linking thermodynamic predictions to putative metabolic behavior. Here, we cultivated biofilms *in situ* on rocks collected from DeMMO, providing a native mixed-mineral surface for biofilm colonization, and mapped biofilm distributions and rock surface chemistry using coupled microscopy/spectroscopy. If biofilm mineral selectivity is occurring, we hypothesize that cell distributions should relate to iron distribution on the rock surface. We use bulk community and spatial analyses to test this hypothesis and constrain biofilm mineral selectivity in this deep continental setting.

## Materials and Methods

### DeMMO

Deep Mine Microbial Observatory is situated in the Sanford Underground Research Facility (SURF) in Lead, South Dakota, United States. SURF, located in the former Homestake Gold Mine, is now dedicated to facilitating subsurface research. DeMMO is comprised of six sites throughout SURF ranging from 244 - 1,478 meters below surface where borehole installations provide access to fracture fluids. Borehole installations are outfitted with ports that allow for aseptic fluid sampling and prevent oxygen from infiltrating the fractures. A detailed description of these installations is available in Osburn et al. (2019). This study focuses on two of these sites, D1 and D3, located at 244 and 610 meters below surface, respectively.

SURF is situated in the Precambrian core of the Black Hills uplift comprised of regionally deformed Paleoproterozoic metasediments cross-cut by Tertiary igneous intrusions. The subsurface infrastructure of SURF is approximately 3.5 km wide and is currently accessible to a depth of 1.5 km; however, previous mining operations extended to a depth of 2.4 km. The SURF mining levels intersect four major rock units: Ellison Formation, Homestake Formation, the upper unit of the Poorman Formation, and the lower Yates Unit within the Poorman Formation. The Ellison Formation is comprised of pelitic phyllite and interbedded quartzite. The Homestake Formation is a gold ore-bearing carbonate-rich iron formation that has been metamorphosed to siderite-rich phyllite and grunerite-rich schist. Gold ore bodies constituting less than 3% by volume of Homestake Formation are rich in sulfide minerals including pyrrhotite and arsenopyrite. The upper unit of the Poorman Formation, hereafter referred to as Poorman Formation, is primarily comprised of graphitic phyllite and carbonate-rich schist locally rich in iron sulfides and large quartz veins overlaying a lower unit of metabasalt (Yates Unit). The minerals that primarily comprise each rock unit are described in Table 1. Tertiary-age rhyolite dikes and associated quartz veins cross-cut the mining levels and mark the latest stage of mineralization in the mine. These veins include the minerals calcite, quartz, pyrite, molybdenite, sphalerite, galena, chalcopyrite, fluorite, gypsum, chlorite, barite, rhodochrosite, and native arsenic (Caddey et al., 1991).

**TABLE 1:** Major minerals present in DeMMO rock units.

	**Ellison**	**Homestake**	**Poorman**	**Yates**
Quartz	X	X	X	
Biotite	X	X	X	
Sericite	X		X	
Pyrrhotite	X	X	X	X
Siderite		X		
Pyrite			X	X
Graphite		X	X	
Grunerite		X		
Chlorite		X		
Calcite			X	X
Ankerite			X	
Dolomite				X
Magnesio-hornblende				X
Oligoclase-andesine				X
Magnetite				X
Titanite				X
Ilmenite				X

We collected cobble-sized (64-256 mm) samples of each of the four major rock units from SURF for field cultivation experiments. Fresh rock was chiseled from the mine tunnel walls and weathered rinds of the rock were removed using a rock saw. Coupons were prepared by slicing ∼2 mm thick, ∼0.5 × 0.5 inch square sections of rock. Coupon surfaces were gradually polished using coarse to fine diamond-impregnated grit grinding disks lubricated with ethanol. We lubricated the grinding disks with ethanol rather than water to mitigate the dissolution of minerals in the rock during polishing. Crushed rock was prepared using a percussion mortar. Coupons and crushed rock were sterilized in a drying oven at 150°C for three days. We chose dry oven sterilization over combustion or autoclaving to mitigate alteration of the minerals (Jenneman and McInerney, 1986; Dezhong et al., 2011).

### Field Cultivation Experiments

We deployed *in situ* colonization reactor experiments containing the four previously described rock units at D1 and D3 in June 2019 and collected the experiments in December 2019. A detailed description of colonization reactor design and construction can be found in Casar et al. (2020). These experiments included crushed native rock mixed with combusted quartz sand to a ratio of ∼1:2 to minimize major changes in pH associated with mineral weathering. Control reactors were filled with only combusted quartz sand and a combusted glass slide. Polished rock coupons or control inert glass slides were included within each reactor for visualizing biofilm distribution and elemental mapping via coupled scanning electron microscopy (SEM) and X-ray energy dispersive spectroscopy (XEDS). Each experiment was carried out in duplicate totaling 10 experiments at each site. Post-incubation, rock coupons and glass slides were fixed in 4% glutaraldehyde in the field and stored at 4°C until preparation for microscopy. Crushed material from experiments was subsampled into sterile tubes and immediately frozen on dry ice in the field for DNA extraction. To compare biofilm communities to planktonic communities, we filtered cells from fracture fluids from boreholes at each site immediately prior to installation and after collection of the reactors. Approximately 1 L of fluid from each site was sampled via sterile tubing connected to sampling ports on borehole installations using *in situ* pressure to force fluids through 0.2 μm Sterivex filters. Filters were immediately frozen on dry ice.

### Microbial Community Analysis

We extracted DNA from crushed rock and sand samples using a Qiagen PowerSoil DNA Isolation Kit and from filters using a MoBIO PowerWater Sterivex DNA Isolation Kit following the manufacturer-suggested protocol. Whole genomic DNA was sent to Argonne National Laboratory for 16S rRNA gene amplicon sequencing of the V4 hypervariable region using 516F (GTGYCAGCMGCCGCGGTAA) and 806R (GGACTACNVGGGTWTCTAAT) universal primers (Caporaso et al., 2011). Paired-end reads were joined, demultiplexed, classified into amplicon sequence variants (ASVs) with the q2-dada2 plugin v 2020.8.0, rarefied, assigned taxonomy, and assessed for alpha diversity with QIIME v 2020.8.0 (Bolyen et al., 2019). We referenced the Silva138 database for taxonomy assignments (Quast et al., 2012). ASVs were rarefied to a depth of 46,822 reads corresponding to the minimum read count among samples in the dataset. We used the NCBI BLAST nucleotide search against the NCBI Refseq database to further identify ASVs of interest. We used the metaMDS function in the vegan package in R for non-metric multidimensional scaling to assess Bray-Curtis dissimilarity among communities at the ASV level. We carried out similarity percentage (SIMPER) analysis to determine taxonomic contribution to between-group Bray-Curtis dissimilarity using the simper function in the vegan R package (Dixon, 2003).

### Spatial Analysis of Biofilm Distribution on Rock Surfaces

We documented biofilm distribution and mapped elements across the surface of 16 rock coupons including each of the four native rock units with replicates from both sites D1 and D3 via coupled SEM/XEDS. We also imaged biofilms on glass slides from control experiments via SEM. In preparation for SEM/XEDS, rock coupons and glass slides were gradually dehydrated in 200 proof ethanol and subjected to critical point drying to preserve cell structure. Coupons and slides were then coated with osmium tetroxide to a thickness of 15 nm in an SPI osmium coater to enhance sample conductivity for imaging. Samples were imaged and mapped using a FEI Quanta 650 environmental SEM with integrated Oxford AZtec EDS/EBSD system in the EPIC facility at the Northwestern University NUANCE center. SEM images were collected at a working distance of 10 mm at a resolution of 1024×1536 pixels and operating voltage of 25 kV. XEDS scans were collected using an energy range of 40 keV divided into 4096 channels for high magnification scans and 2048 channels for low magnification scans with a 40μs pixel dwell time.

Bulk element compositions for each rock unit were determined by averaging 8 XEDS scans at 70x magnification collected randomly across rock coupon surfaces. We probed for correlations between taxa and bulk element composition of rock used in the respective experiments using the cor function in the stats R package and visualized these relationships using the heatmaply R package (R Core Team, 2013; Galili et al., 2018). Taxa included in the correlation analysis are those comprising at least 1% of a community and present in at least half of all communities for a given site.

We targeted iron-rich minerals in rock coupons for high resolution spatial analyses, mapping elements and biofilm distributions around these minerals at a scale where microbes are detectable. To accomplish this, we collected SEM/XEDS data from one transect per coupon covering ∼0.025 mm^2^ at a magnification of 3000x crossing a mineral grain boundary into the background matrix. Additionally, we collected 5 random SEM images at 3000x from control glass slides.

To document cell distributions in SEM images, individual images from each transect were stitched into panoramic images in Photoshop using the photomerge tool and biofilm features were manually curated. In Photoshop, pixels associated with cells or fungal morphologies were painted using the paintbrush tool and these layers were exported as TIFFs. Cells include rod, coccoid, and non-bifurcating filamentous morphologies typical of bacteria and archaea, while fungal morphologies include bifurcating filaments that resemble fungal hyphae with local spore-like structures (Supplementary Figure 1). To couple the stitched SEM and XEDS images, the coordinates for individual SEM images in each rock transect were exported from Photoshop and used for stitching XEDS images into panoramic raster images using the Raster package in R (Hijmans and Van Etten, 2012). XEDS panoramic raster images were then stacked into raster bricks and merged with rasterized layers of SEM and biofilm feature panoramic images. Transect maps including element and biofilm feature distributions were generated from the raster bricks using the ggplot2 and RasterVis packages in R and standard element colors referenced from Jmol (J Team, 2007; Wickham, 2009; Perpiñán and Hijmans, 2021).

To probe for visual differences among biofilms, we summarized biofilm feature properties in Photoshop including area and circularity calculated as 4π(area/perimeter^2^). Circularity is the degree of roundness, where cocci will be more circular than rods or filaments. Cell morphology counts were generated from circularity where “filament”, “rod”, or “cocci” classifications correspond to a circularity of ≤0.1, 0.1–0.7, or >0.7, respectively. The percent of transect area covered by cell or fungal morphologies was calculated by dividing the total pixel area of cells or fungal morphologies by the total transect area.

To determine if regions of high cell densities correspond toiron-rich regions, we estimated cell 2D kernel densities from cellcentroid coordinates and visualized densities as contours withggplot2. Cell centroids were calculated from biofilm featuresconverted from raster to polygon using the sf package in R (Pebesma, 2018). To determine the dependence of cell distributions on element distributions, we fit Poisson point process models to the data using the spatstat package in R (Baddeley and Turner, 2005). Poisson point process models are appropriate for modeling spatial count data over a continuous space where the intensity varies according to environmental conditions, common in ecological studies (Renner et al., 2015). We probed for elements that explain cell distribution by comparing nested models, e.g., simple null random models vs. more complex models with covariates, using the ANOVA likelihood ratio test in the stats R package. The null models assume that the cell distributions are random, whereas models with covariates assume cell distributions can be explained by the underlying element distributions. Cell positions were represented as points using centroid coordinates, whereas element distributions were represented by raster images. We generated complex models with up to 5 combinations of elements present in the rock surface. The best models were chosen from each dataset based on computed likelihood ratio test statistics: the lowest *p*-value, highest deviance, and fewest number of covariates, where *p*-values are ≤0.05 indicating statistical significance of the chi-square distribution goodness-of-fit test. The deviance here is defined as the difference between the deviance from the simple null model and that of the alternate complex model, e.g., deviance(null) – deviance(complex). Positive, larger deviances correspond to a better fit to the more complex model with covariates relative to the simple null model, while a deviance of 0 indicates that the complex model is as good as the null model. We simulated point process models by estimating the inhomogeneous L-function, Besag’s transformation of the Ripley’s reduced second moment K_r_ function, with Monte Carlo simulation envelopes from cell point patterns both with and without covariates using the Linhom function in spatstat. Observed distributions that fall within the simulation envelopes indicate that the model is a good fit.

## Results

### Rock Properties

**FIGURE 1:**
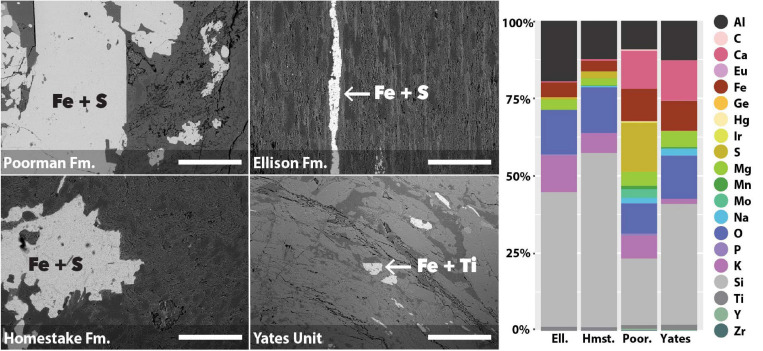
SEM images of rock coupons representing the four major rock units at DeMMO with arrows pointing to examples of mineral grains selected for spatial analyses **(left)**; images were collected at 105x magnification, scale bars represent 500 μm. Bulk chemistry of the four major rock units at DeMMO **(right)**.

We used coupled SEM/XEDS to map element compositions across the four major rock units at DeMMO: Poorman Formation, Homestake Formation, Ellison Formation, and Yates Unit. We determined bulk rock chemistry by averaging a series of 70x magnification XEDS scans across each rock type (Figure 1). The four rock types are each dominantly comprised of silicon, aluminum, and oxygen, as is typical of silicate rocks. Poorman Formation and Yates Unit are more iron, calcium, and sodium-rich than Ellison and Homestake formations, consistent with their more mafic characters. Of the four rock types, Poorman Formation has the highest sulfur content, whereas Yates Unit is sulfur-poor. Poorman Formation also contains small amounts of molybdenum, zirconium, iridium, and yttrium. We targeted iron-rich minerals on rock surfaces for high resolution spatial analyses (Figure 1), including iron and sulfur-rich grains present in Poorman, Ellison, and Homestake formation coupons and iron and titanium-rich grains present in Yates Unit coupons.

### Bulk Community Diversity

**FIGURE 2:**
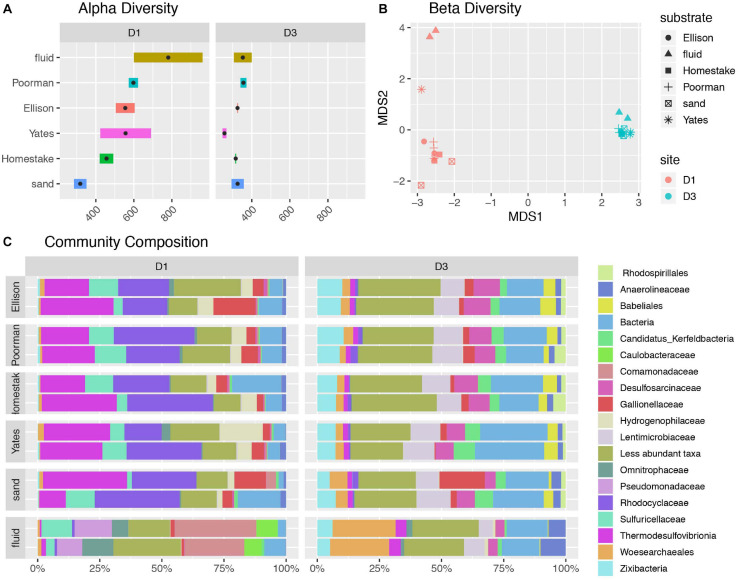
Diversity of communities at DeMMO. **(A)** Alpha diversity as the number of observed ASVs at a sequencing depth of 46,500 reads. Black points represent mean observed ASV values. **(B)** Beta diversity as NMDS ordination of ASVs. **(C)** Community composition as taxa identified to the family level for biofilms in duplicated reactor experiments sampled in December 2019 and fracture fluids sampled in June and December 2019. Less abundant taxa are those representing less than 5% of communities.

A comparison of alpha diversity shown as phylogenetic diversity, Shannon diversity, and number of observed ASVs reveals fluid communities are more diverse than biofilms, and rock-hosted biofilms are more diverse than biofilms on inert controls at D1 (Figure 2A and Supplementary Figure 2). D1 fluid communities are the most diverse with 781 ASVs on average. The highest diversity observed among D1 communities across rock types is in experiments using Yates Unit, averaging 598 ASVs, compared to the other rock units ranging from 456-555 ASVs. D1 biofilms on inert sand are least diverse, averaging 381 ASVs. In contrast, D3 communities exhibit comparable diversity across all fluids and biofilms and are generally less diverse overall than D1 communities, ranging from 326-353 ASVs on average.

A taxonomic comparison of microbial communities reveals that within each site, biofilm and fluid communities share phyla but in very different abundances and are distinguished at lower taxonomic levels, while rock-hosted vs. control biofilm communities exhibit no clear differences in community composition to the family level (Figure 2C). Here we define dominant taxa as those that comprise at least 10% of a community. D1 fluid communities are dominated by Caulobacteraceae within the Alphaproteobacteria, Pseudomonadaceae, Sulfuricellaceae and Comamonadaceae within the Gammaproteobacteria, and Omnitrophaceae within the Verrucomicrobiota, while biofilms are dominated by Sulfuricellaceae, Rhodocyclaceae Hydrogenophilaceae, and Gallionellaceae within the Gammaproteobacteria and Thermodesulfovibrionia within the Nitrospirota. The D3 fluid communities are dominated by Woes archaeales within the Nanoarchaeota, Zixibacteria, and Anaerolinaceae within the Chloroflexi, while biofilms are dominated by Zixibacteria, Desulfosarcinaceae within the Desulfobacterota, Gallionellaceae within the Gammaproteobacteria, and Lentimicrobiaceae within the Bacteriodota. Two candidate groups are enriched (up to ∼5%) in D3 biofilms relative to fluid communities including Candidatus Kerfeldbacteria within the Patescibacteria and Babeliales within the Dependentiae.

Sulfuricellaceae and Hydrogenophilaceae at both sites are dominantly members of the genera *Sulfuricella* and *Thiobacillus*, respectively. Among D1 biofilms, *Thiobacillus* are enriched in rock-hosted communities relative to controls, comprising as much as 17.2% biofilms on Yates Unit. Unassigned taxa within the domain Bacteria comprise as much as 19.9% of D1 communities and 27.8% of D3 communities. Although abundant, these unassigned taxa are primarily comprised of only one ASV with 85.9% identity at 98% coverage to *Desulfotomaculum hydrothermale* strain Lam5.

The ASV-level NMDS ordination reflects trends in alpha and beta diversity, where fluid communities occupy a distinct ordination space separate from biofilm communities at each site (Figure 2B). D1 rock-hosted biofilm communities are separated from inert control communities, in contrast to D3 rock-hosted and control biofilm communities that plot very closely in NMDS space. A SIMPER analysis of D1 rock vs. control biofilm communities reveals that the taxa contributing to the top 75% of average between-group dissimilarity are dominantly comprised of ASV’s within the class Thermodesulfovibrionia (Supplementary Figure 3).

### Taxonomic Correlation With Bulk Rock Chemistry

**FIGURE 3:**
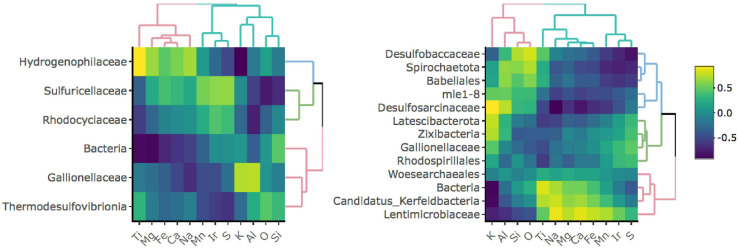
Correlation matrices between taxa and bulk rock chemistry for D1 **(left)** and D3 **(right)** communities. Only taxa that comprise at least 1% of and are present in at least 50% of communities for a given site are shown.

To investigate whether specific taxa correlate with elements comprising iron-bearing minerals present in the rock, e.g., Fe, S, or Ti, we carried out a correlation analysis between bulk rock chemistry and rock-hosted biofilm communities classified to the family level (Figure 3). D1 taxa form three distinct clades comprised of Hydrogenophilaceae (blue clade), Sulfuricellaceae and Rhodocyclaceae (green clade), and unclassified Bacteria, Gallionellaceae, and Thermodesulfovibrionia (red clade). Hydrogenophilaceae correlate most positively with Ti and Fe and are anti-correlated with S. Taxa within the green clade correlate most positively with S and are moderately positively correlated with Fe. Taxa within the red clade correlate most positively with K, Al, O, and Si. D3 taxa form three distinct clades comprised of Desulfobaccaceae, Spirohaetota, Babeliales, mle1-8, and Desulfosarcina (blue clade), Latescribacterota, Zixibacteria, Gallionellaceae, and Rhodospirillales (green clade), and Woes archaeales, unclassified Bacteria, Candidatus Kerfeldbacteria, and Lentimicrobiaceae (red clade). Taxa within the blue clade correlate most positively with K, Al, Si, and O and are moderately positively correlated with Ti. Taxa within the green clade correlate most positively with S. Taxa within the red clade correlate most positively with Ti and Fe and are moderately positively correlated with S.

### Biofilm Properties

**FIGURE 4:**
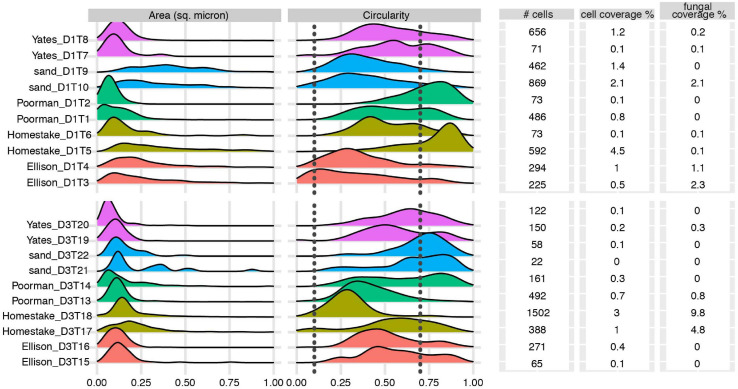
Biofilm summary statistics: Cell area and circularity distributions, total number of observed cells, cell and fungal coverage over the transect area for each coupon. Sample IDs correspond to substrate type and coupon ID. Vertical dashed lines represent circularity thresholds for filaments, rods, and cocci (0-0.1, 0.1-0.7, and 0.7-1, respectively). X-axes are truncated at a maximum value of 1.

**FIGURE 5:**
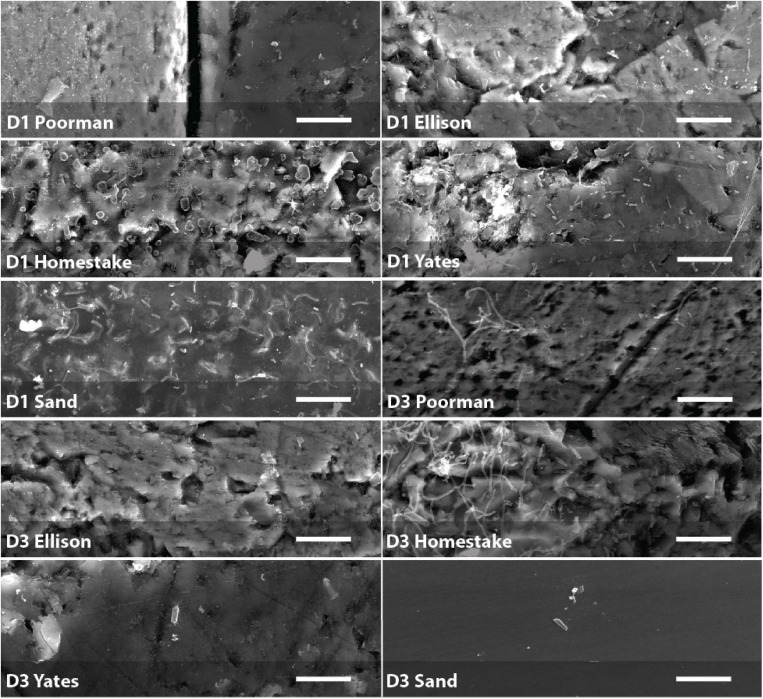
Biofilms on D1 and D3 rock coupons and glass slides from control experiments with sand. Scale bars represent 10 μm.

We documented biofilm properties using high-resolution SEM images taken from transects across iron and sulfur-rich mineral grain boundaries in rock coupons and glass slides in control experiments with sand (Figure 1). Biofilms are comprised of mono-layered cells and differ in their physical properties across study sites and across the four rock units and inert controls (Figures 4, 5 and Supplementary File 1). The following reported cell sizes represent minimums due to potential shrinkage during sample preparation. Cell sizes across rock-hosted biofilms are predominantly ∼0.1 μm^2^ while biofilms on control glass slides exhibit a wider range of cell sizes from ∼0.1-0.8 μm^2^. Cell morphologies are dominantly rods across all biofilms with the exception of biofilms on D1 Homestake and Poorman formation and D3 sand which are dominantly cocci. The total number of cells observed across each rock transect range from ∼70-1,500 corresponding to coverages ranging from ∼1-4.5% of the transect area. In some samples, we observe ‘fungal morphologies’ defined here as features that are bifurcating with round features at the ends resembling fungal hyphae with spores (Supplementary Figure 1), covering as much as ∼10% of the total transect area in D3 biofilms on Homestake Formation.

### Cell Distribution as a Function of Rock Chemistry

**FIGURE 6:**
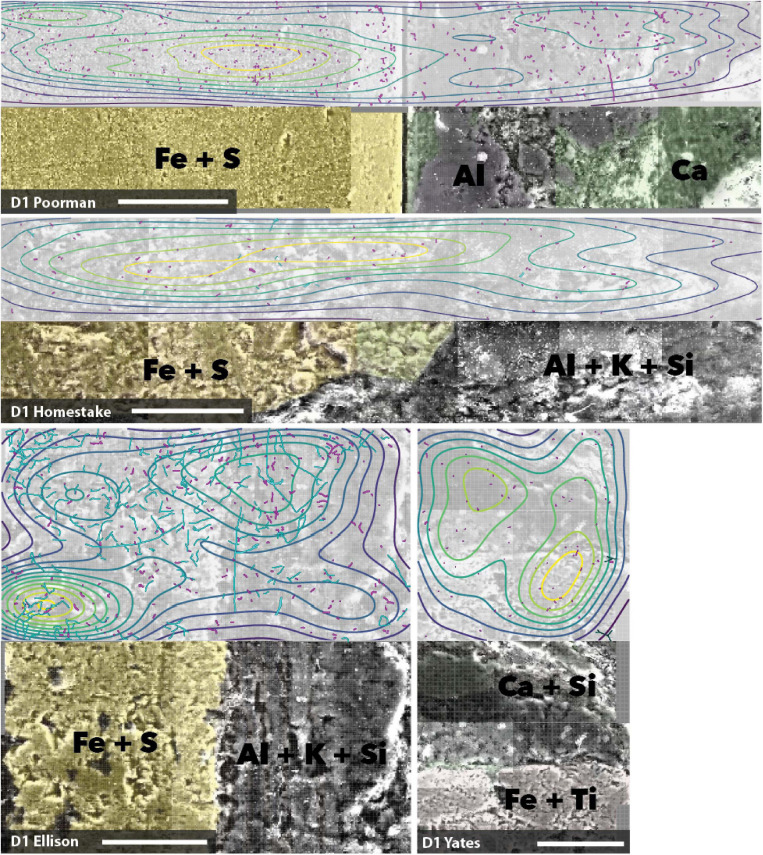
Coupled SEM/XEDS images of D1 biofilms on native rock coupons from selected experiments. Top panels for each rock type show cell (magenta) and fungal feature (cyan) distribution with kernel density contours where yellow represents the highest density, bottom panels show coupons colored by elements. Transects are stitched images collected at 3000x magnification. Scale bars represent 50 μm.

**FIGURE 7:**
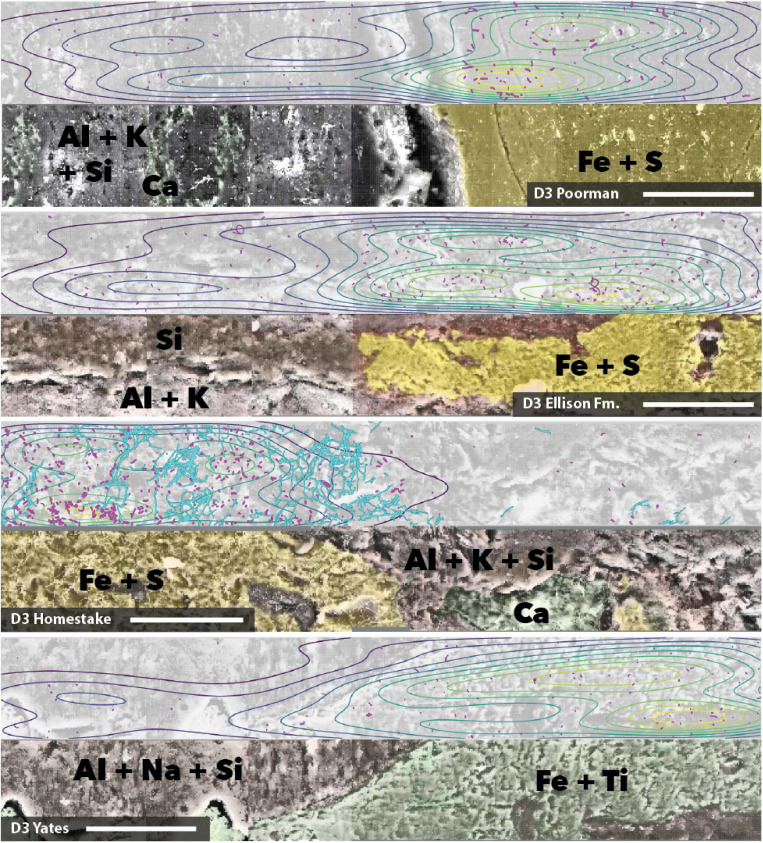
Coupled SEM/XEDS images of DeMMO3 biofilms on native rock coupons from selected experiments. Top panels for each rock type show cell (magenta) and fungal feature (cyan) distribution with kernel density contours where yellow represents the highest density, bottom panels show coupons colored by elements. Transects are stitched images collected at 3000x magnification. Scale bars represent 50 μm.

We mapped cell and element distributions using high resolution SEM/XEDS transects across targeted mineral grain boundaries (Figure 1). This data was used in spatial analyses to determine the relationship between cell distributions and rock surface chemistry. To probe for biofilm hotspots, we performed 2D kernel density estimates on cells revealing that peak densities occur on sulfur, iron, and titanium-rich regions of rock surfaces (Figures 6, 7 and Supplementary File 2).

To determine whether cell distributions are spatially dependent upon element distributions in the rock surfaces, we fit Poisson point process models to the data. In all transects where the number of observed cells exceeded ∼70, the complex models with covariates fit the data better than the null random models. The best fit models, defined here as those with the lowest significant *p*-values (≤0.05), fewest number of covariates, and highest deviances, reveal that sulfur, titanium, and iron are the most frequently invoked covariates for improving the model fit (Table 2). In some cases, the best models included only one covariate, as in coupon D1T6 Homestake Formation modeled with sulfur, whereas in other cases the best model saturated the maximum number of covariates we allowed, as in coupon D3T19 Yates Unit modeled with As, Ca, Er, Fe, and S. Nevertheless, every significant model includes Fe, S, or Ti. In three experiments with Poorman and Ellison formations (coupons D1T2, D3T13, and D3T15), the total numbers of observed cells were very low (∼70 total cells) and the complex models with covariates did not fit the data better than the null random model.

**TABLE 2:** Poisson point process model likelihood ratio test statistics.

**Substrate**	**Coupon ID**	**Model**	***p*-value**	**Deviance**
Poorman	D1T1	Ca + S	2.88 × 10^–8^	34.73
Ellison	D1T3	Ca + Mn + Ti	0.04	8.08
Ellison	D1T4	S	0.02	5.34
Homestake	D1T5	Fe + Mg + Mn + S	1.55 × 10^–35^	169.10
Homestake	D1T6	S	0.01	6.29
Yates	D1T7	Al + K + Ti	4.06 × 10^–4^	18.17
Yates	D1T8	Al + Na + Ti + W + Y	0.02	13.90
Poorman	D3T14	Mn + Na + S + Ti	1.32 × 10^–9^	47.30
Ellison	D3T16	S + Si	1.66 × 10^–14^	63.46
Homestake	D3T17	Fe + Mn + P + S + Si	1.77 × 10^–32^	158.82
Homestake	D3T18	Al + Hg + S	0.001	16.03
Yates	D3T19	As + Ca + Er + Fe + S	0.004	17.44
Yates	D3T20	Fe + Na	4.45 × 10^–5^	20.04

To simulate the fitted point process models, we estimated the inhomogeneous L-function. The observed distributions occur within the Monte-Carlo simulated envelopes for the estimates for the majority of the relevant range of distances (Supplementary File 3). These simulations reveal no clear deviation of point patterns from model predictions and therefore indicate good fit, with the exception of D1T5 and D3T17 Homestake Formation experiments.

## Discussion

### Biofilm Community Composition and Characteristics

We probed community compositions among fluid and biofilm communities to investigate environmental drivers of community differences. If mineral selectivity is occurring, we expect rock-hosted communities to differ from glass or fluid-hosted communities. Within each study site, biofilm community composition is consistent across rock types and glass controls to the family level. While host rock mineral composition can drive major differences in biofilm community composition, these differences may not be measurable at the bulk community scale. For example, communities cultivated *in situ* on single crystal pyrite were enriched in members of the Gallionellaceae and Desulfobulbaceae, representing as much as ∼20% of the community (Casar et al., 2020). However, in this study the bulk community composition aggregates cubic centimeters of rock, including a variety of minerals, and therefore is unlikely to capture mineral-specific trends. We acknowledge the potential for primer bias against archaea with the use of universal primers (Baker et al., 2003); however, we do not expect that our primer selection is significantly distorting our view of archaeal diversity given previous whole metagenome sequencing did not reveal an archaea-dominated system (Momper et al., 2017). Nevertheless, we do observe the enrichment of taxa known to form biofilms in D1 and D3 biofilm communities, including members within the Rhodocyclaceae, Gallionellaceae, and Hydrogenophilaceae (Lütters-Czekalla, 1990; Bosch et al., 2012; Chu and Wang, 2013; Jangir et al., 2016).

Our community data and SEM observations suggest some variability within putative eukaryotic members of the biofilm communities. We observed enhanced abundances of fungal morphologies on some sample surfaces over others, particularly on Homestake and Ellison formation coupons. These features are long, thin, and bifurcating with large round features on the ends in some samples, resembling fungal hyphae with spores (Supplementary Figure 1). Additionally, we observed putative amoebal symbionts, e.g., members of the order Babeliales (Yeoh et al., 2016), enriched in D3 biofilm communities. Our 16S rRNA gene survey captures only prokaryotic DNA, thus eukaryotic fungal or amoebal communities are not represented in our data and we do not focus on their contribution to biofilm diversity here.

While we observe no major differences among biofilm communities to the family level, an NMDS ordination of communities at the ASV-level distinguishes rock-hosted communities from controls at D1. This statistical separation between rock-hosted and control biofilms is supported by stark visual differences in cell morphologies between rock coupons and glass slides, where biofilms on glass have widely varying cell sizes relative to biofilms on rock surfaces. Although no major differences in circularity distributions (i.e. rod vs. cocci-dominated) are apparent, our SEM observations reveal visual differences between biofilms on rock vs. biofilms on glass controls, where cells are generally larger in D1 control biofilms. While morphology cannot provide taxonomic information, it can be useful for probing for relative differences between biofilms in parallel experiments, where differences between cell morphologies may signal a phylogenetic or phenotypic response to attachment surface chemistry. If we assume no major taxonomic differences among cells of varying sizes, then we could interpret morphological differences as being a survival response to the environment (Justice et al., 2008). Otherwise if taxonomic compositions represent major differences, then morphology could reflect phylogenetic differences driven by environmental selection (Siefert’t and Fox, 1998). In either case, morphology can be used as an indicator of differences that we can attribute to the rock composition because all other variables for a set of parallel experiments should be the same. Taxa that contribute the most to the average between-group dissimilarity among rock-hosted vs. glass-hosted biofilms are those within the class Thermodesulfovibrionia. There is no clear trend in average abundances of Thermodesulfovibrionia ASVs between rock or glass substrates - some are more abundant on rock and some are more abundant on glass. Due to the lack of classification beyond the class level for these ASV’s it is difficult to speculate on what metabolic characteristics could be driving these differences in abundance. However, taxa within this class are broadly capable of iron and sulfur-cycling (Spring et al., 1993; Henry et al., 1994), and community differences could be driven by differences in iron or sulfur-cycling capacity. Overall, our bulk community data suggests that biofilm-forming taxa at DeMMO may be generalists capable of growth on solid substrates where available.

Correlation analysis between rock-hosted biofilm communities and bulk rock chemistry further probes geochemical drivers of community compositions. At D1, the Hydrogenophilaceae correlate most strongly with the elements Fe and Ti that comprise mineral grains targeted in Yates rock, while the Sulfuricellaceae correlate most strongly with Fe and S that comprise mineral grains targeted in Poorman, Ellison, and Homestake rock. The Hydrogenophilaceae and Sulfuricellaceae are dominated by taxa within the genera *Thiobacillus* and *Sulfuricella*, respectively. *Thiobacillus* are known iron and sulfur cyclers, and *Sulfuricella* are known sulfur-cyclers, thus these correlations may be reflective of the metabolic capacities of these taxa. At D3, the taxa that correlate most strongly with Fe, S, and Ti include candidate groups Zixibacteria, Latescibacteria, and Candidatus Kerfeldbacteria in which metabolisms have not yet been well-established, thus these correlations may shed light on their potential for metal cycling at DeMMO.

### Spatial Distribution of Biofilms Suggests Mineral Selectivity

Our spatial analyses reveal biofilm hotspots and putative mineral selectivity occurs on Fe and S, or Fe and Ti-bearing minerals. We visualized biofilm distributions using 2D kernel density estimates, indicating peak densities coincide with Fe and S, or Fe and Ti-rich regions. To probe the spatial dependence among cells and elements detected across rock surfaces, we carried out spatial modeling. We compared nested Poisson point process models, e.g., null random models and complex models with element covariates. Across all samples where cell observations were sufficiently high, the addition of Fe, S, and Ti to Poisson point process models significantly improves model fit to the observed cell distributions and the majority of model simulations indicate good fit. These models suggest cell distributions can be explained primarily by the distributions of Fe, S, and Ti, which we interpret as a signal of preferential colonization of the iron sulfide or iron titanium minerals, putatively in the form of ferrous iron-bearing pyrite, or pyrrhotite and ilmenite present throughout DeMMO (Caddey et al., 1991).

In some cases, models fit poorly to cell distributions or did not perform better than the null random models, which we attribute to low quality data. Biofilms from samples from two experiments with Homestake Formation were characterized by cells much larger than those observed on other coupons, and model simulations indicate cells are significantly dispersed after accounting for the underlying element distribution. Poor model fit in this case may be an artifact of using cell centroids that poorly represent the large area covered by these cells. In samples where the total number of observed cells was very low, the addition of covariates did not improve the model fit and thus no spatial dependence upon the underlying element distribution was apparent. This is most likely due to insufficient point data for the transect area where cell distributions are too sparse to model.

Nevertheless, our interpretation of mineral selectivity in the majority of our experiments is supported by previous thermodynamic models and growth experiments at DeMMO indicating that dissimilatory ferrous iron oxidation of a variety of ferrous iron-bearing minerals is thermodynamically favorable. While ilmenite was not specifically modeled, ferrous iron in ilmenite may be a favorable electron donor for energy metabolisms in DeMMO biofilm communities. Nitrate-dependent pyrite oxidation where sulfur is oxidized to sulfate is highly thermodynamically favorable at DeMMO and *in situ* growth experiments with single crystal pyrite yielded orders of magnitude higher biomass relative to fluid and inert control communities at D1 and D3 (Casar et al., 2020). In the present study, we observed the enrichment of *Thiobacillus* in rock-hosted biofilms at D1, a genera known to selectively colonize pyrite as well as use pyrite as a growth substrate (Ohmura et al., 1993; Bosch et al., 2012). Taken together, our observations suggest metabolically-driven mineral selectivity is driving biofilm distribution at DeMMO.

### Implications of Mineral Selectivity for Biomass Estimates and Biogeochemical Cycles

Mineral transformations at scale in the continental subsurface have the potential to play an important role in subsurface biogeochemical cycles. One such potentially important transformation is the dissolution of pyrite, the most abundant sulfide mineral in Earth’s crust and a prevalent mineral throughout DeMMO. The present and previous studies suggest nitrate-dependent iron sulfide oxidation may be an important metabolic process at DeMMO with the potential to support a significant amount of biofilm biomass. Here, cell densities of 10^6^ cells/cm^2^ on pyrite are possible, and may exceed cell densities that are four orders of magnitude greater than the associated planktonic communities (Casar et al., 2020). In the present study, D1 biofilms are enriched in *Thiobacillus*, a genus known to carry out nitrate-dependent pyrite oxidation (Bosch et al., 2012). Nitrate-dependent pyrite oxidation releases aqueous ferrous iron and sulfate and can release trace metals such as As (Yan et al., 2019), a trace metal present as arsenopyrite at DeMMO (Caddey et al., 1991) which we detected in high resolution XEDS scans of the major rock units in the present study. We previously detected elevated levels of sulfate and ferrous iron as well as trace metals such as As in fracture fluids sampled from D1 and D3, where fracture fluids seeping from the mine tunnel walls into the oxidizing atmosphere support large and thick *Gallionella ferruiginea* biofilms comprised of twisted iron oxide stalks (Osburn et al., 2019). The fracture fluid chemistry is reflective of host rock mineralogy throughout DeMMO; however the mechanism of mineral dissolution that produces the observed chemistry is unclear. The present study suggests extensive bioleaching of iron and sulfur-bearing minerals by biofilms may help explain DeMMO fracture fluid chemistry.

The relative importance of biofilm-driven biogeochemical transformations in the continental subsurface is a function of total biofilm biomass. The most recent estimate of biofilm biomass in the subsurface is between 20-80% of the total biomass. These estimates are based on models extrapolated over large regions that incorporate crust type (e.g., craton), temperature, density, porosity, depth, and direct cell counts on rock cores, sediments, and fluids (Magnabosco et al., 2018; Flemming and Wuertz, 2019). Lithology was not previously found to correlate to continental subsurface cell concentrations from sediments and rock cores; however, these included lithologies such as ‘metamorphic’, ‘metasediment’, and ‘subglacial sediment’ that don’t provide mineralogical context (Magnabosco et al., 2018). Our experiments demonstrate the potential for highly heterogeneous biofilm distribution on rock surfaces as a function of mineral selectivity. At DeMMO, iron-bearing minerals are important drivers of biofilm distribution and growth, supporting hotspots of biomass relative to the surrounding matrix. At a regional scale, this could have important implications for biomass estimates; e.g., an iron-rich basalt deposit could host significantly more biomass than a silica-rich quartzite deposit. Currently there is a lack of data regarding biofilm biomass as it relates to mineralogy in continental subsurface settings, and large uncertainties surround biofilm contributions to biomass here. Our findings warrant refined continental subsurface biomass estimates that account for host rock mineralogy as more data becomes available in the future.

## Conclusion

We probed mineral selectivity by biofilm communities in the continental deep subsurface to gain insight into potential drivers of biomass and biogeochemical cycles here. Our experiments suggest that iron and sulfur-rich minerals drive biofilm colonization at DeMMO. Spatial analyses of biofilm distribution suggest their dependence upon the distribution of iron, sulfur, and titanium in the rock surface, which we attribute to iron sulfide minerals including pyrite and pyrrhotite and the iron titanium mineral ilmenite prevalent throughout DeMMO. Biofilms on native rock were enriched in taxa capable of nitrate-dependent pyrite oxidation, a thermodynamically favorable reaction here that releases aqueous ferrous iron and sulfate. The elevated levels of ferrous iron and sulfate detected in fracture fluids suggest bioleaching of iron and sulfur-bearing minerals is likely a volumetrically important process at DeMMO when considered at the kilometer scale. Given the geological ubiquity of iron and sulfur-rich minerals and magnitude of the biomass they can support, these mineral transformations are likely important for subsurface biogeochemical cycles. Current estimates of continental subsurface biofilm biomass range widely and do not incorporate host rock mineralogy. As more data becomes available in the literature, we suggest that future modeling efforts should evaluate host rock mineralogy as a key variable driving biomass distribution in deep continental settings.

## Data Availability Statement

All code and corresponding data used in this study are available at github.com/CaitlinCasar/Casar2021_DeMMO_RockHostedBiofilms. This Targeted Locus Study project has been deposited at DDBJ/ENA/GenBank under the accession KEQC00000000.

## Author Contributions

CC designed the study, collected the samples, deployed field experiments, performed microscopy and analyses, and wrote the manuscript. BK was the field manager of the project and directed each field expedition. MO advised this research. All authors contributed to the creation of the manuscript and study design.

## Conflict of Interest

The authors declare that the research was conducted in the absence of any commercial or financial relationships that could be construed as a potential conflict of interest.
