# Supplementary material for: Rock-Hosted Subsurface Biofilms: Mineral Selectivity Drives Hotspots for Intraterrestrial Life
Source: Front Microbiol. 2021 Apr 9;12:658988. doi: 10.3389/fmicb.2021.658988 (PMC8062869; doi:10.3389/fmicb.2021.658988)
Supplement: Supplementary file 2 [file Image_1.PDF]

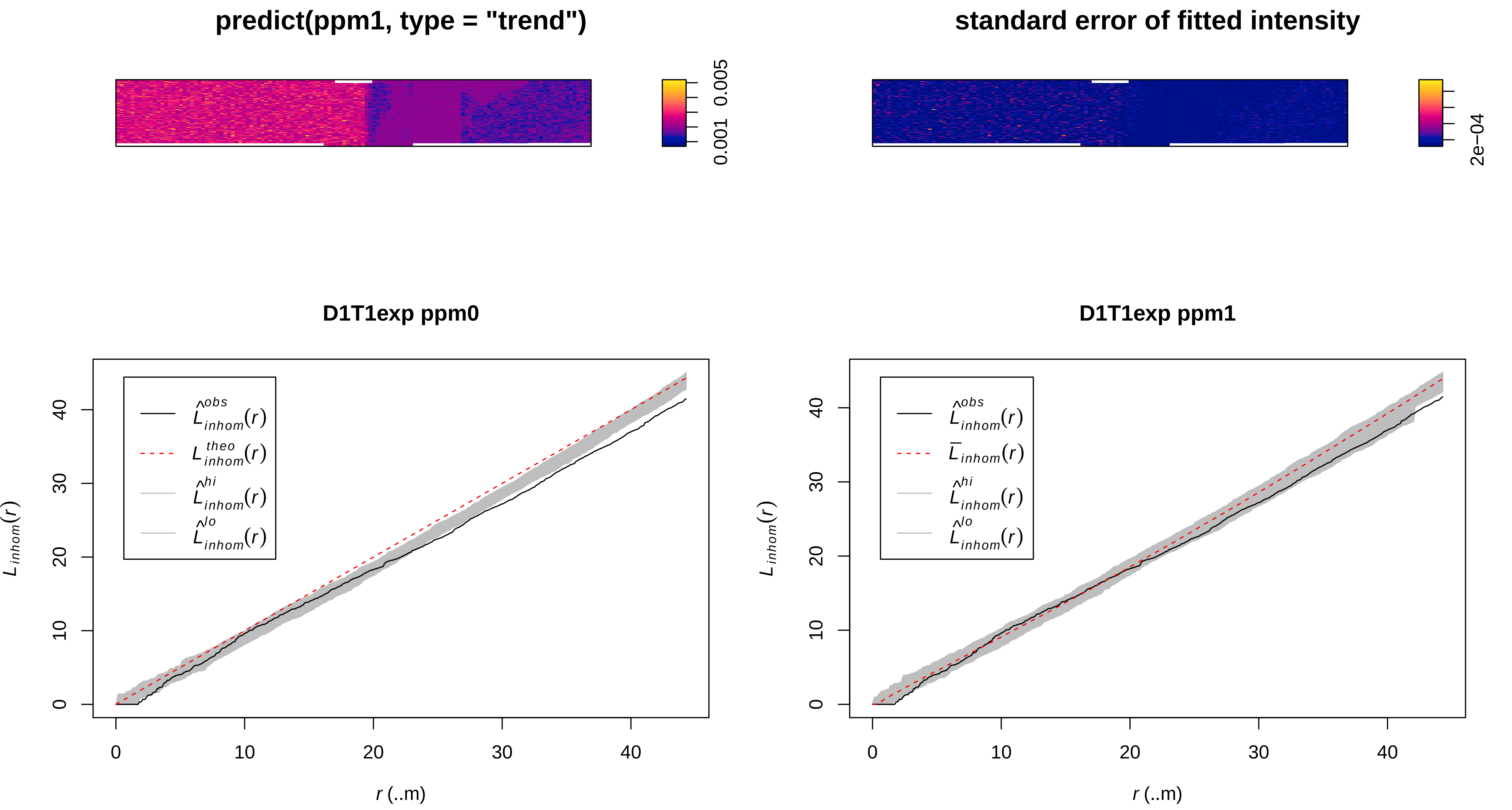

predict(ppm1, type = "trend")
0.001 0.005
standard error of fitted intensity
2e−04
D1T1exp ppm0
D1T1exp ppm1
L inhom(r)
r (..m)
0
10
20
30
40

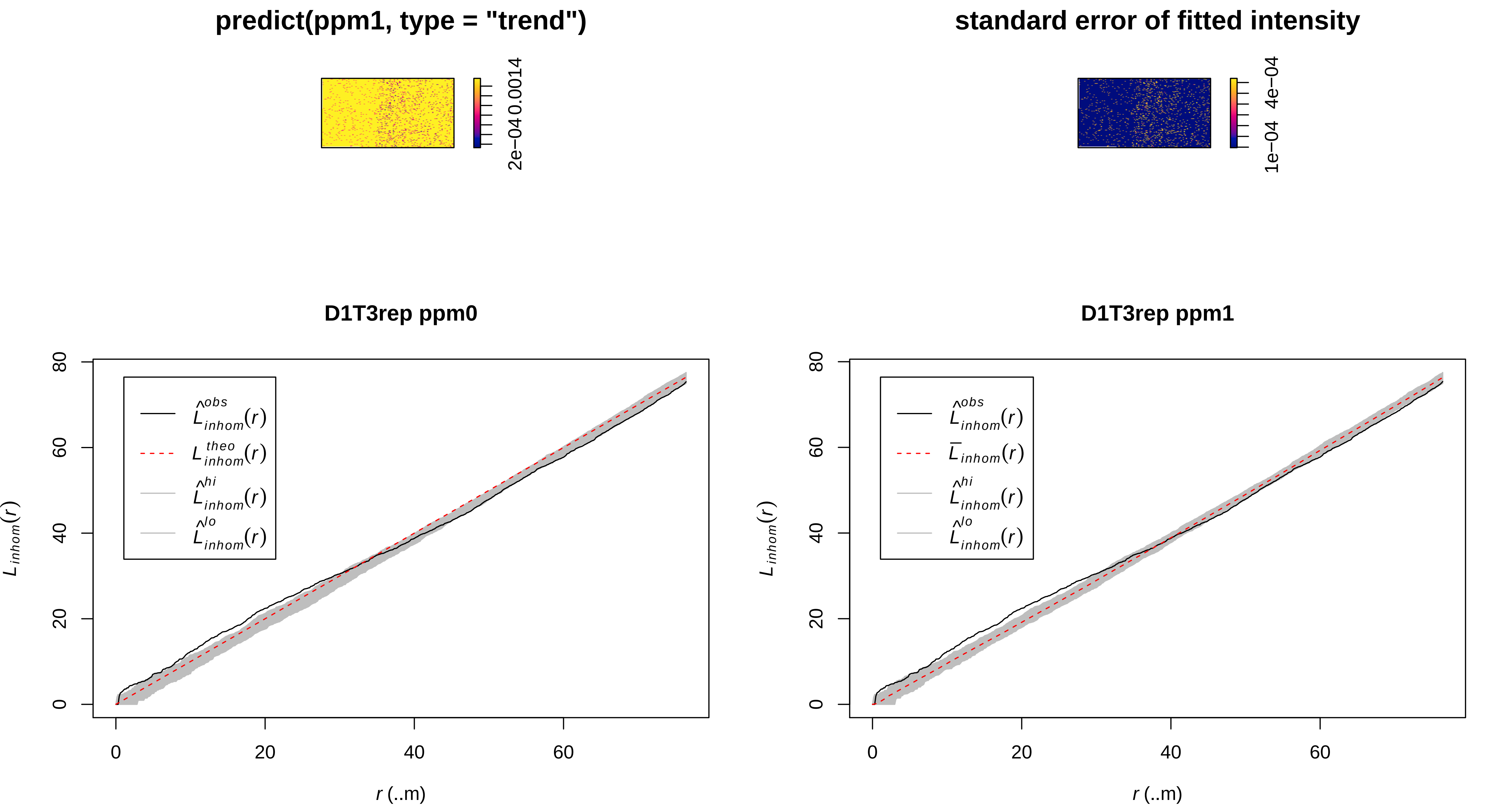
predict(ppm1, type = "trend")
2e−04
0.0014
standard error of fitted intensity
1e−04
4e−04
D1T3rep ppm0
D1T3rep ppm1
$L_{inhom}(r)$
r (..m)
$\hat{L}^{obs}_{inhom}(r)$
$L^{theo}_{inhom}(r)$
$\bar{L}_{inhom}(r)$
$\hat{L}^{hi}_{inhom}(r)$
$\hat{L}^{lo}_{inhom}(r)$
0
20
40
60
80

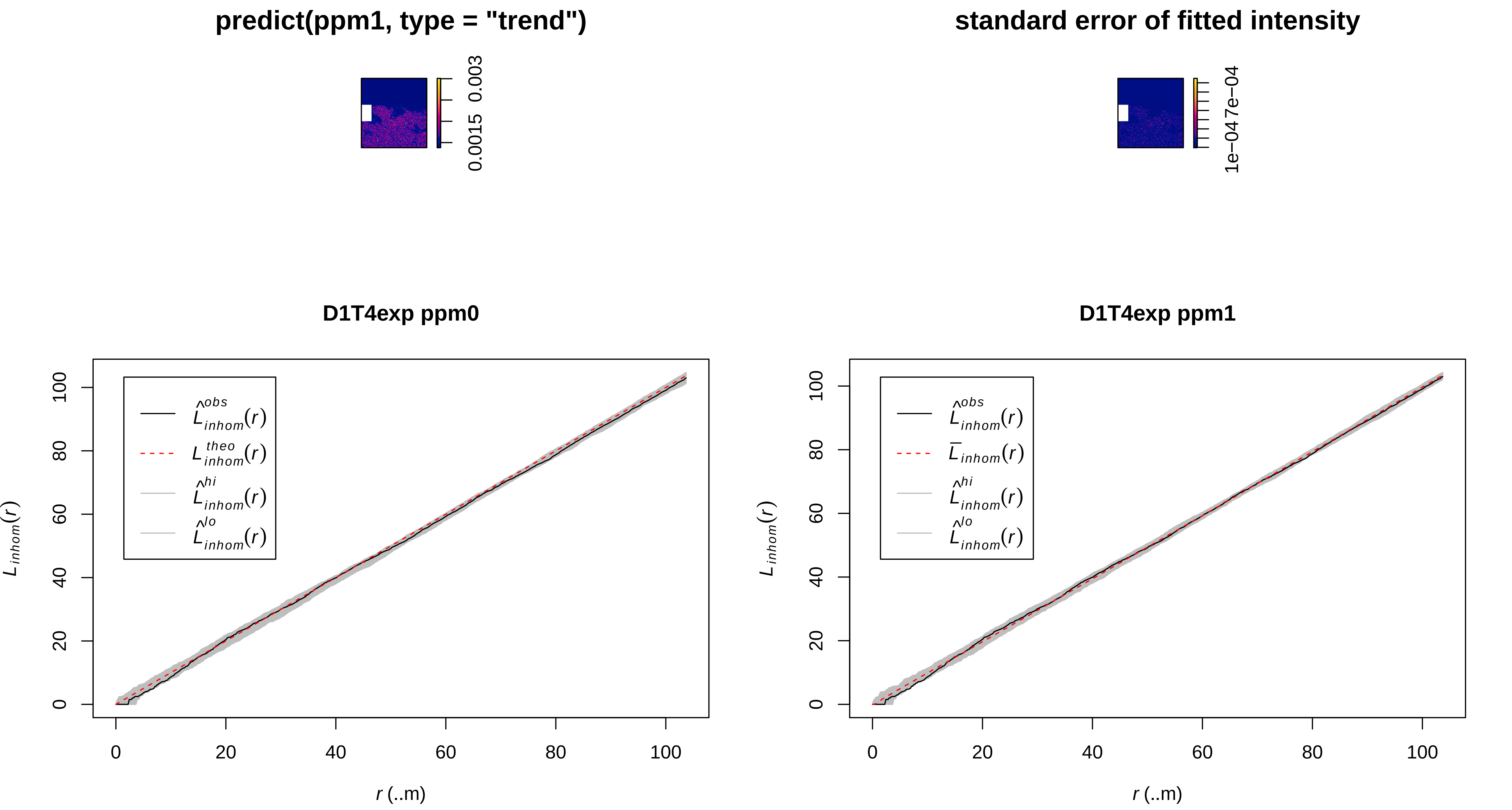
predict(ppm1, type = "trend")
0.0015 0.003
standard error of fitted intensity
1e−04 7e−04
D1T4exp ppm0
D1T4exp ppm1
$\hat{L}^{obs}_{inhom}(r)$
$L^{theo}_{inhom}(r)$
$\bar{L}_{inhom}(r)$
$\hat{L}^{hi}_{inhom}(r)$
$\hat{L}^{lo}_{inhom}(r)$
$L_{inhom}(r)$
r (..m)
0
20
40
60
80
100

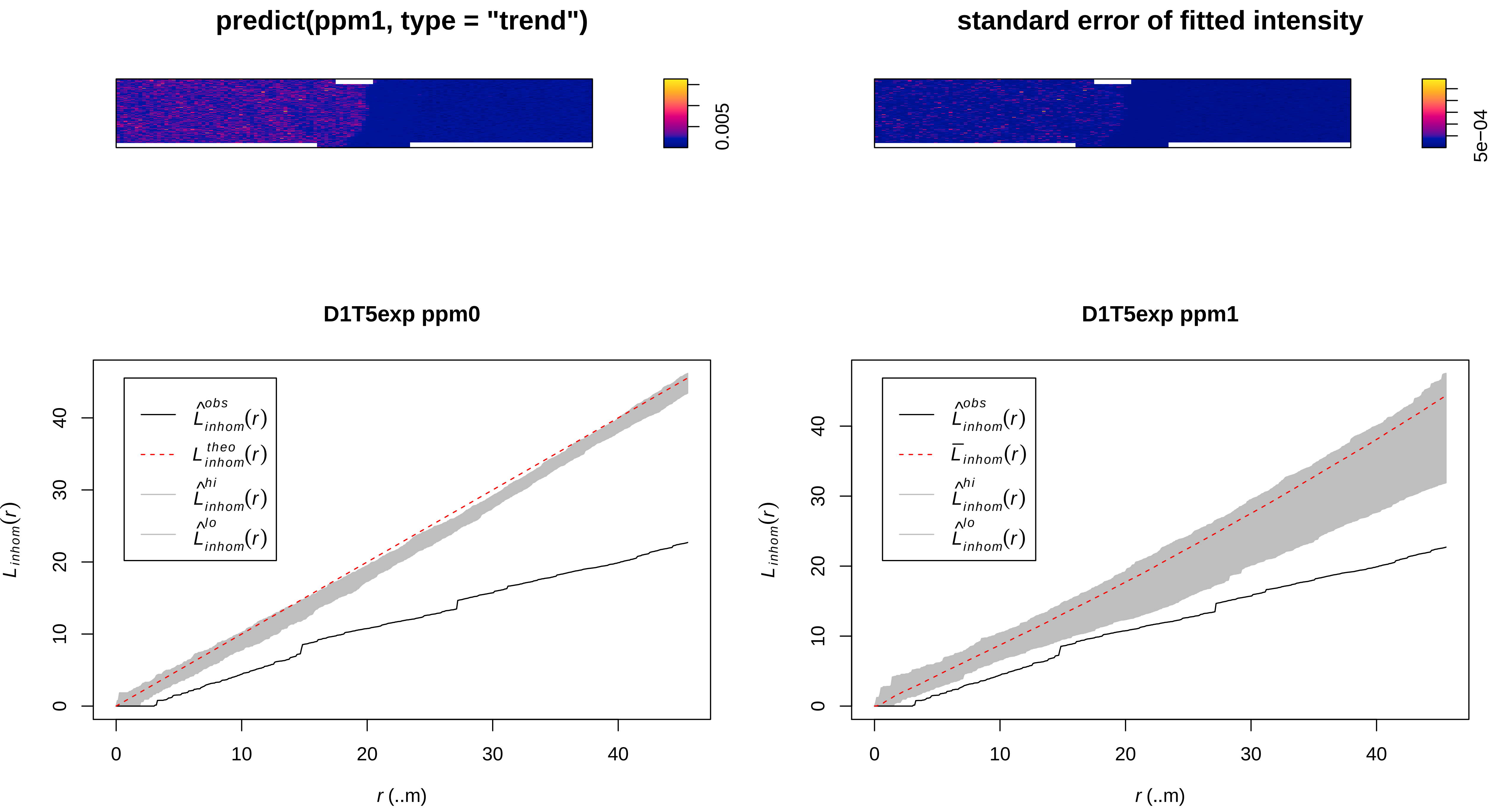

predict(ppm1, type = "trend")
0.005
standard error of fitted intensity
5e−04
D1T5exp ppm0
D1T5exp ppm1
$\hat{L}^{obs}_{inhom}(r)$
$L^{theo}_{inhom}(r)$
$\bar{L}_{inhom}(r)$
$\hat{L}^{hi}_{inhom}(r)$
$\hat{L}^{lo}_{inhom}(r)$
$L_{inhom}(r)$
r (..m)
0
10
20
30
40

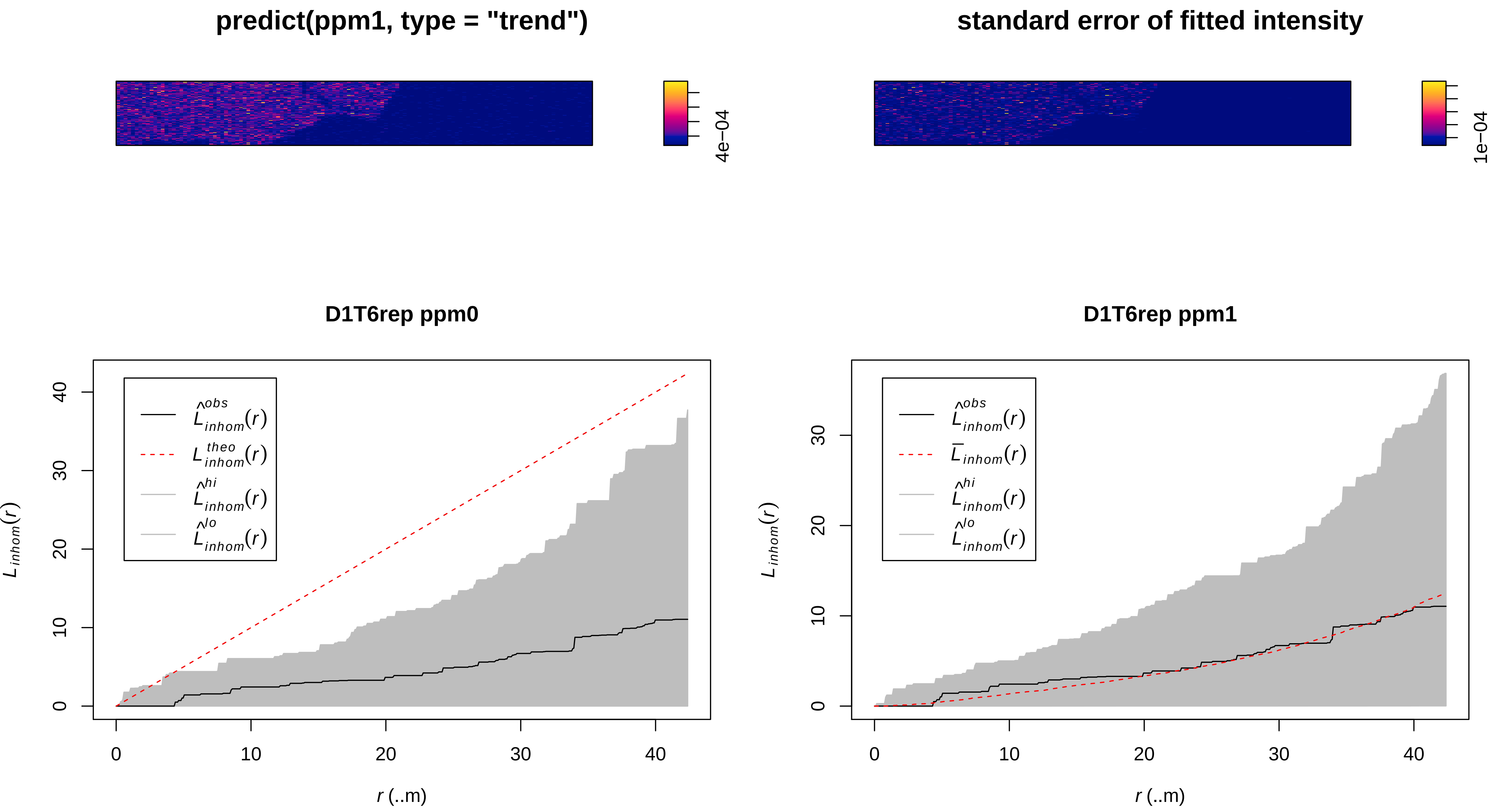

predict(ppm1, type = "trend")
4e−04
standard error of fitted intensity
1e−04
D1T6rep ppm0
$\hat{L}^{obs}_{inhom}(r)$
$L^{theo}_{inhom}(r)$
$\hat{L}^{hi}_{inhom}(r)$
$\hat{L}^{lo}_{inhom}(r)$
$L_{inhom}(r)$
0
10
20
30
40
r (..m)
D1T6rep ppm1
$\hat{L}^{obs}_{inhom}(r)$
$\bar{L}_{inhom}(r)$
$\hat{L}^{hi}_{inhom}(r)$
$\hat{L}^{lo}_{inhom}(r)$
$L_{inhom}(r)$
0
10
20
30
40
r (..m)

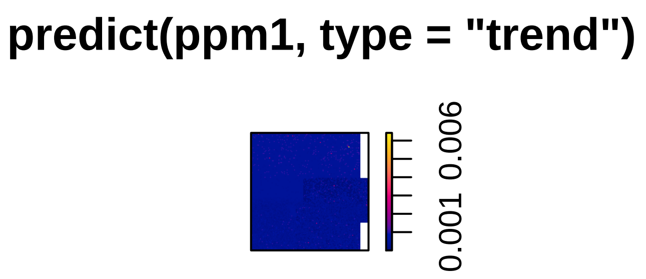
predict(ppm1, type = "trend")
0.001 0.006

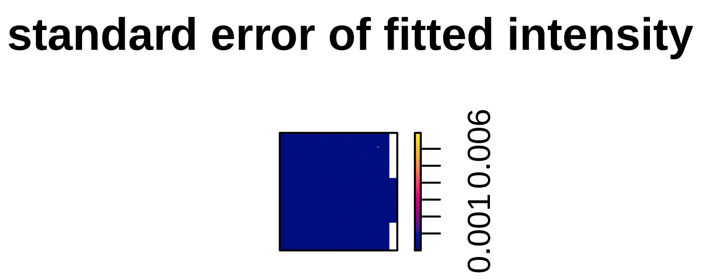
standard error of fitted intensity
0.001 0.006

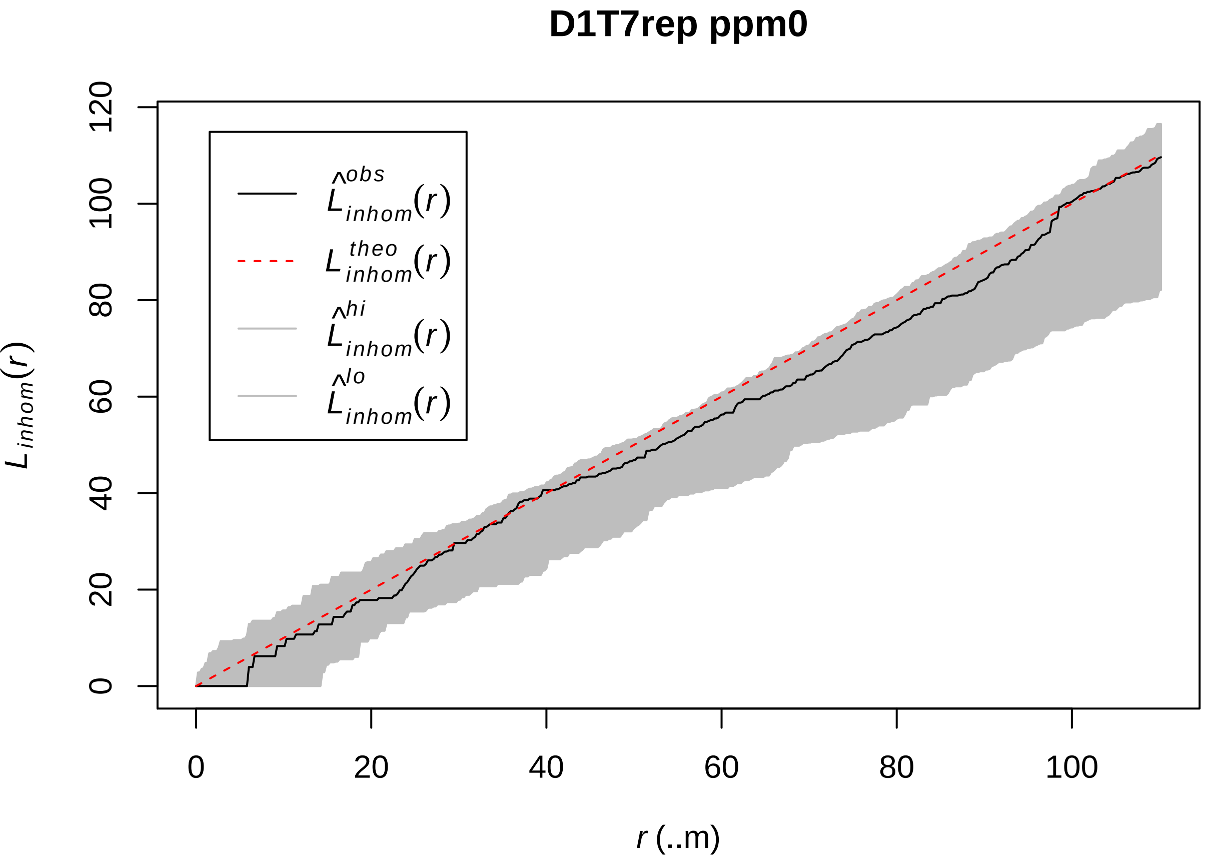
D1T7rep ppm0
$\hat{L}^{obs}_{inhom}(r)$
$L^{theo}_{inhom}(r)$
$\hat{L}^{hi}_{inhom}(r)$
$\hat{L}^{lo}_{inhom}(r)$
$L_{inhom}(r)$
r (..m)
0 20 40 60 80 100
0 20 40 60 80 100 120

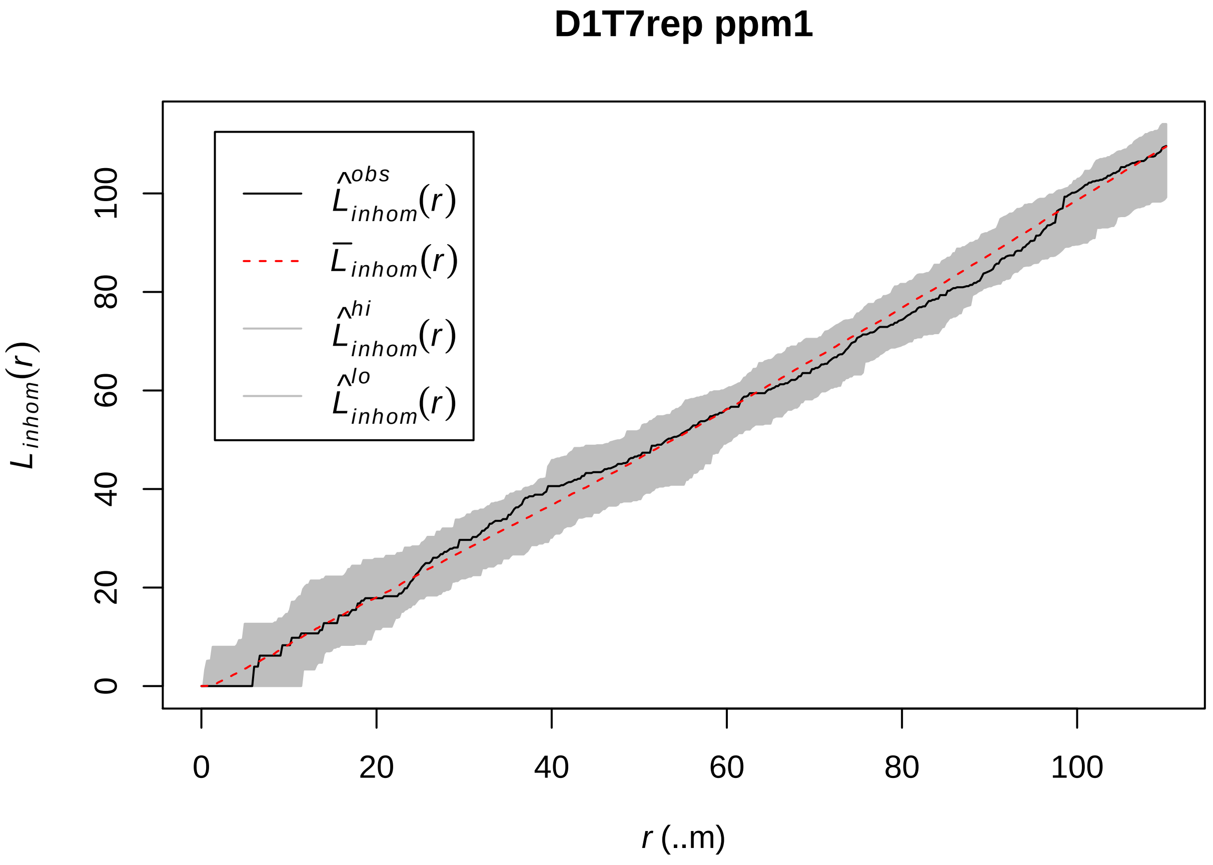
D1T7rep ppm1
$\hat{L}^{obs}_{inhom}(r)$
$\bar{L}_{inhom}(r)$
$\hat{L}^{hi}_{inhom}(r)$
$\hat{L}^{lo}_{inhom}(r)$
$L_{inhom}(r)$
r (..m)
0 20 40 60 80 100
0 20 40 60 80 100

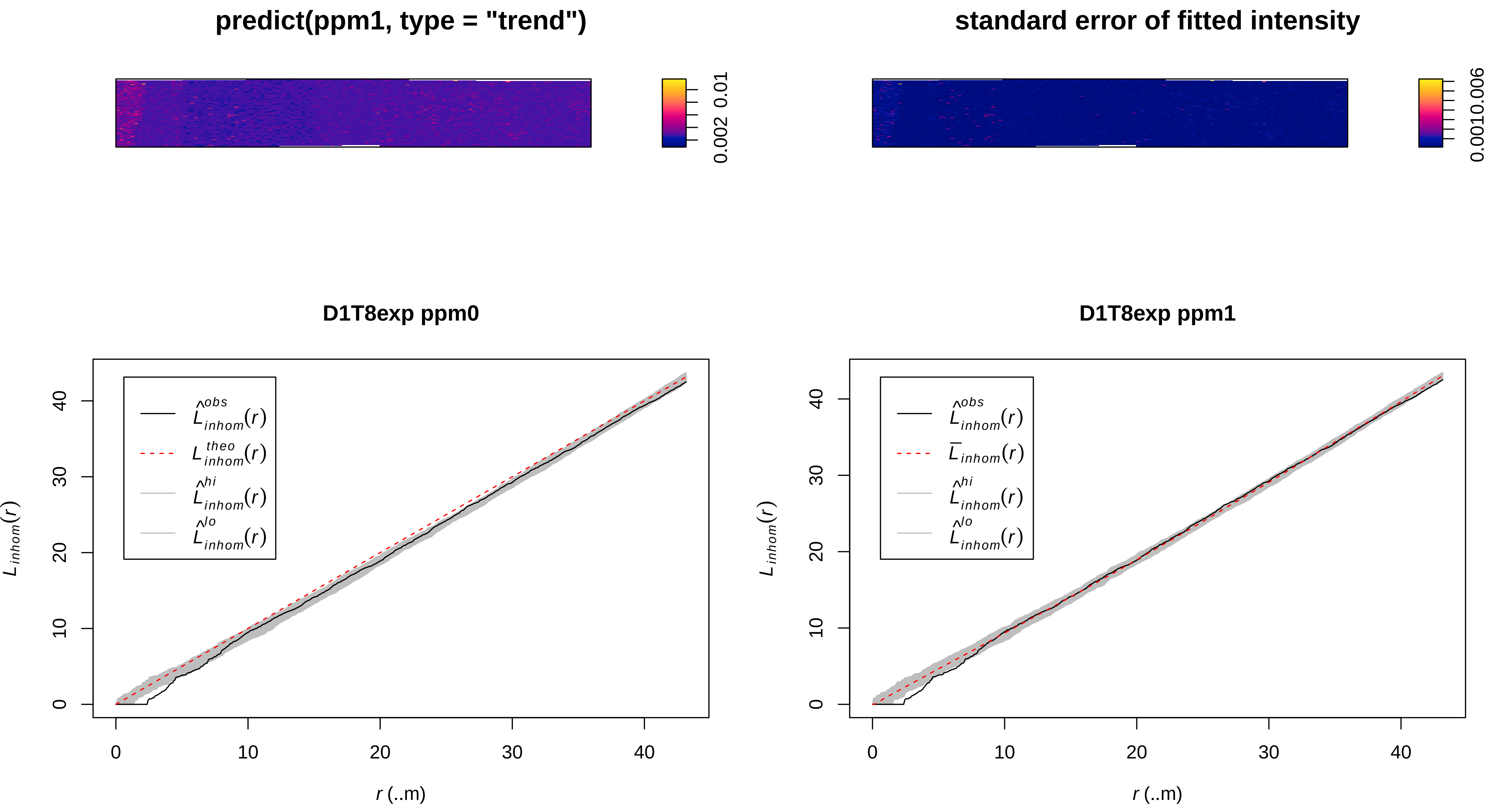

predict(ppm1, type = "trend")
0.002 0.01
standard error of fitted intensity
0.001 0.006
D1T8exp ppm0
D1T8exp ppm1
$L_{inhom}(r)$
r (..m)
$\hat{L}^{obs}_{inhom}(r)$
$L^{theo}_{inhom}(r)$
$\hat{L}^{hi}_{inhom}(r)$
$\hat{L}^{lo}_{inhom}(r)$
$\bar{L}_{inhom}(r)$
0
10
20
30
40

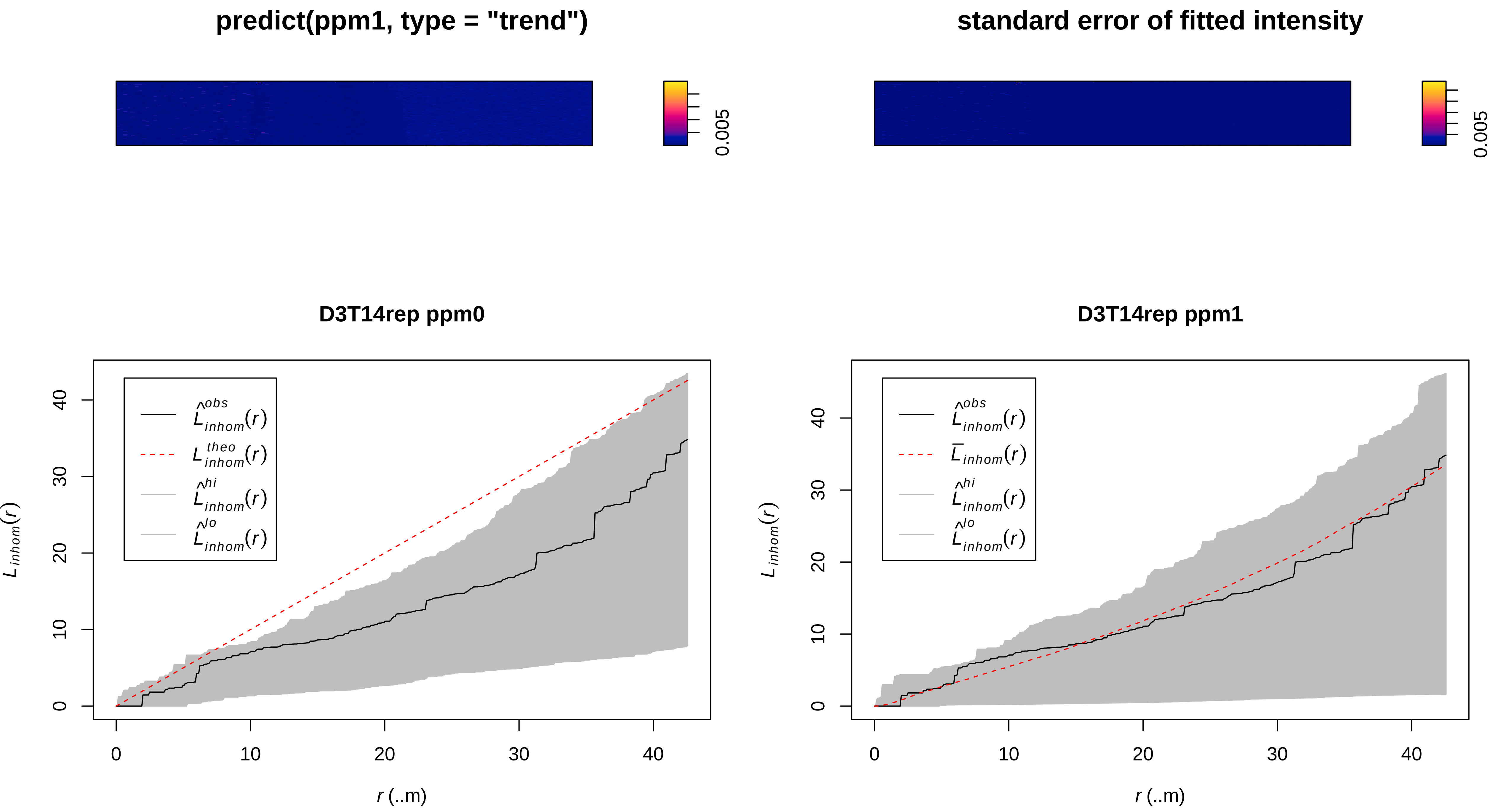

predict(ppm1, type = "trend")
0.005
standard error of fitted intensity
0.005
D3T14rep ppm0
$\hat{L}^{obs}_{inhom}(r)$
$L^{theo}_{inhom}(r)$
$\hat{L}^{hi}_{inhom}(r)$
$\hat{L}^{lo}_{inhom}(r)$
$L_{inhom}(r)$
r (..m)
D3T14rep ppm1
$\hat{L}^{obs}_{inhom}(r)$
$\bar{L}_{inhom}(r)$
$\hat{L}^{hi}_{inhom}(r)$
$\hat{L}^{lo}_{inhom}(r)$
$L_{inhom}(r)$
r (..m)

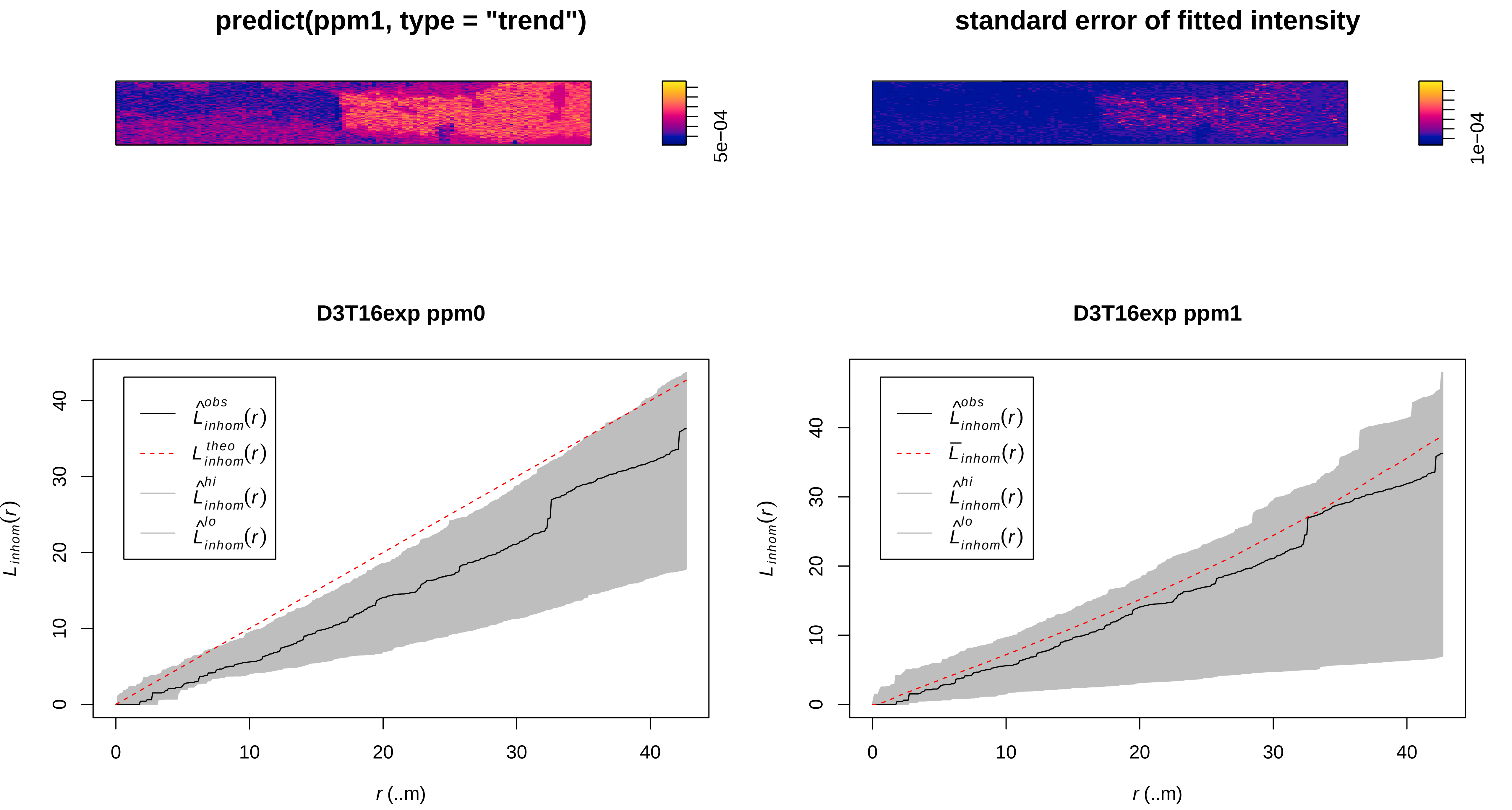
predict(ppm1, type = "trend")
5e−04
standard error of fitted intensity
1e−04
D3T16exp ppm0
D3T16exp ppm1
$\hat{L}^{obs}_{inhom}(r)$
$L^{theo}_{inhom}(r)$
$\hat{L}^{hi}_{inhom}(r)$
$\hat{L}^{lo}_{inhom}(r)$
$\bar{L}_{inhom}(r)$
$L_{inhom}(r)$
r (..m)
0
10
20
30
40

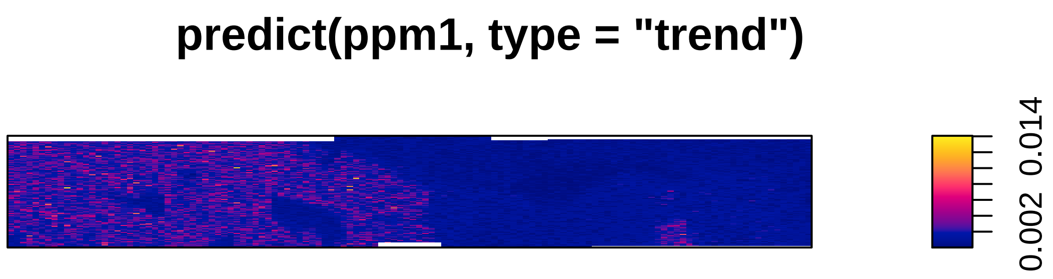
predict(ppm1, type = "trend")
0.002 0.014

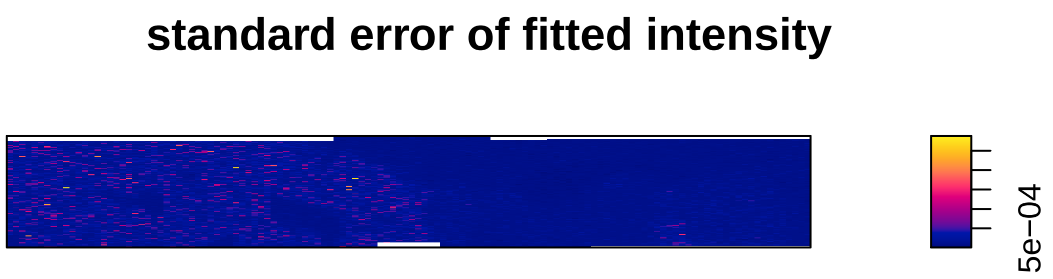
standard error of fitted intensity
5e−04

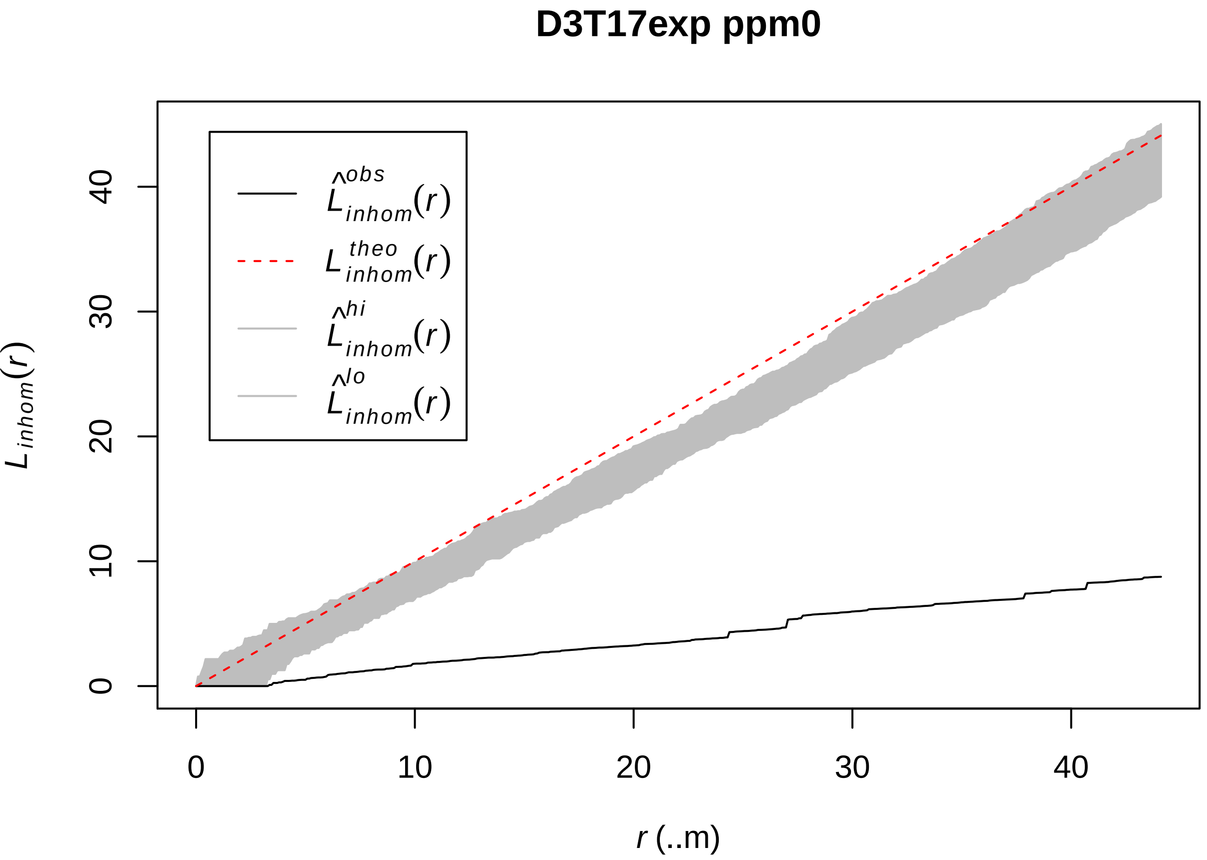
D3T17exp ppm0
$\hat{L}^{obs}_{inhom}(r)$
$L^{theo}_{inhom}(r)$
$\hat{L}^{hi}_{inhom}(r)$
$\hat{L}^{lo}_{inhom}(r)$
$L_{inhom}(r)$
r (..m)
0
10
20
30
40

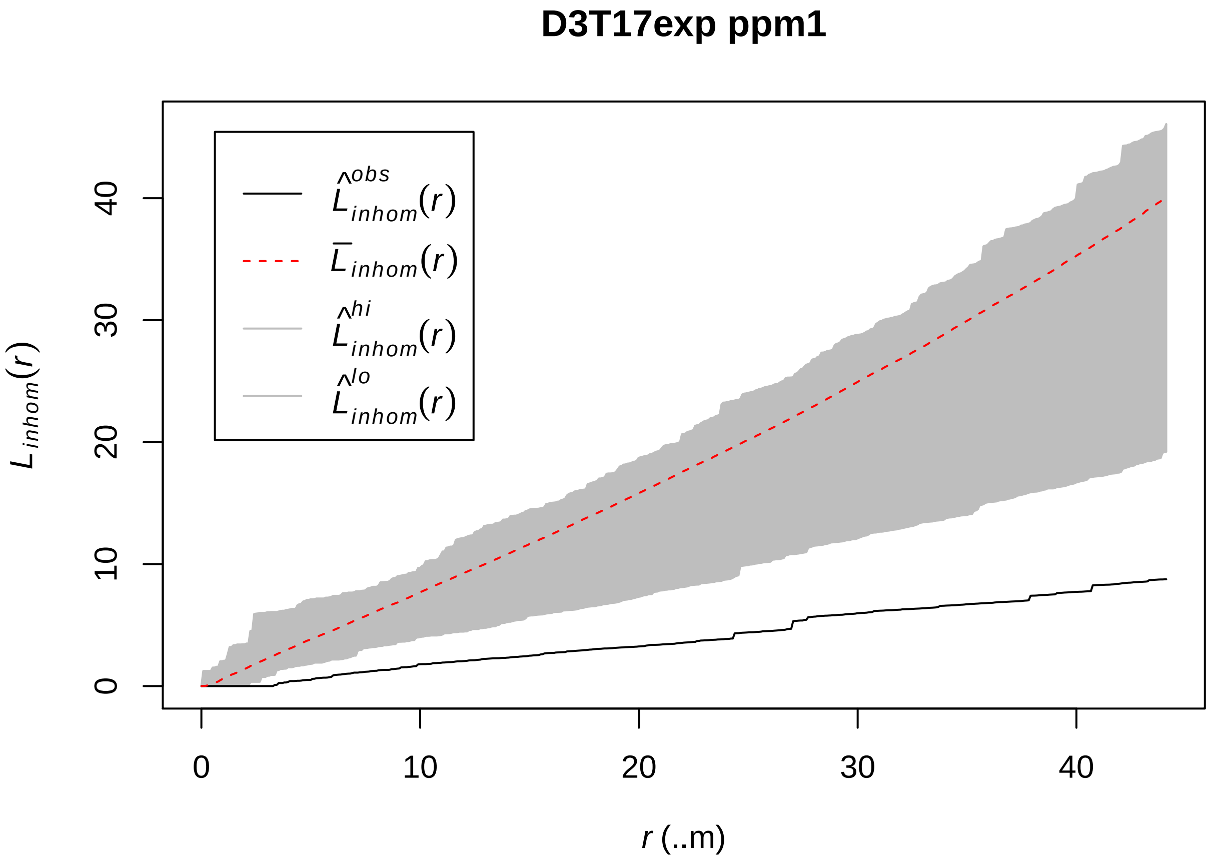
D3T17exp ppm1
$\hat{L}^{obs}_{inhom}(r)$
$\bar{L}_{inhom}(r)$
$\hat{L}^{hi}_{inhom}(r)$
$\hat{L}^{lo}_{inhom}(r)$
$L_{inhom}(r)$
r (..m)
0
10
20
30
40

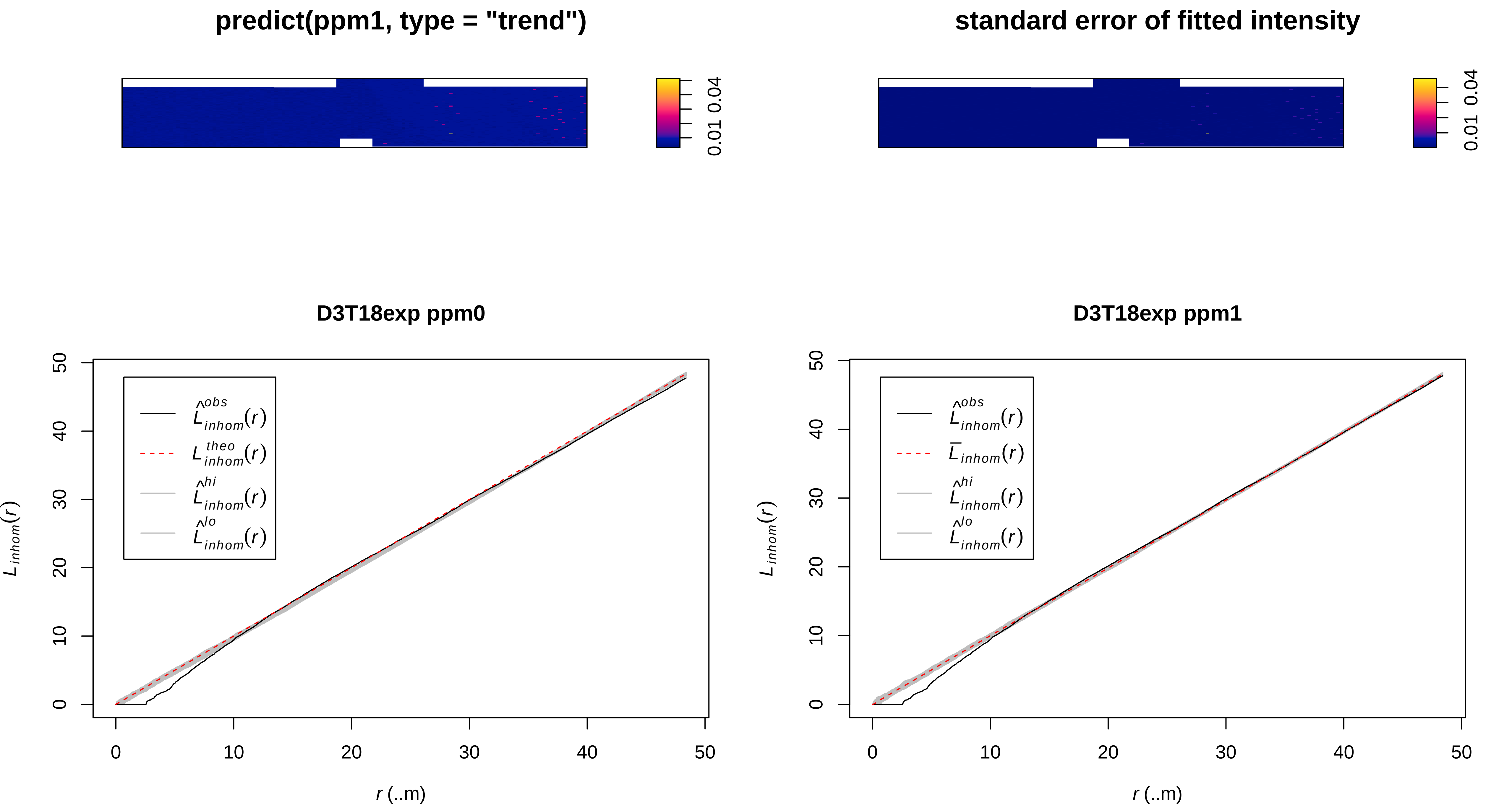

predict(ppm1, type = "trend")
0.01 0.04
standard error of fitted intensity
0.01 0.04
D3T18exp ppm0
D3T18exp ppm1
$L_{inhom}(r)$
r (..m)
0
10
20
30
40
50
$\hat{L}^{obs}_{inhom}(r)$
$L^{theo}_{inhom}(r)$
$\bar{L}_{inhom}(r)$
$\hat{L}^{hi}_{inhom}(r)$
$\hat{L}^{lo}_{inhom}(r)$

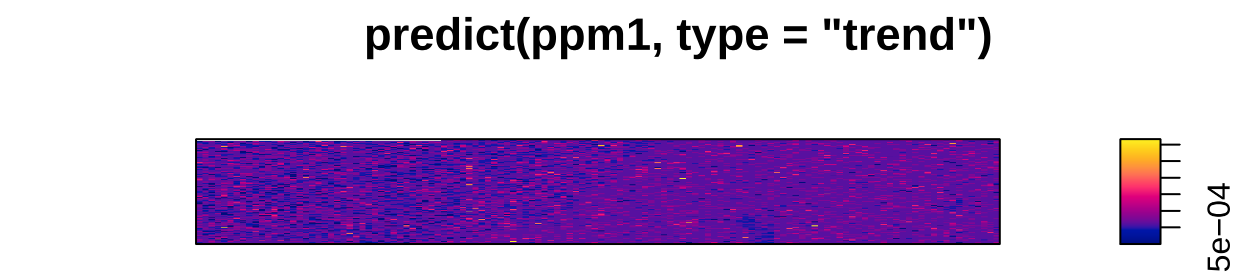
predict(ppm1, type = "trend")
5e−04

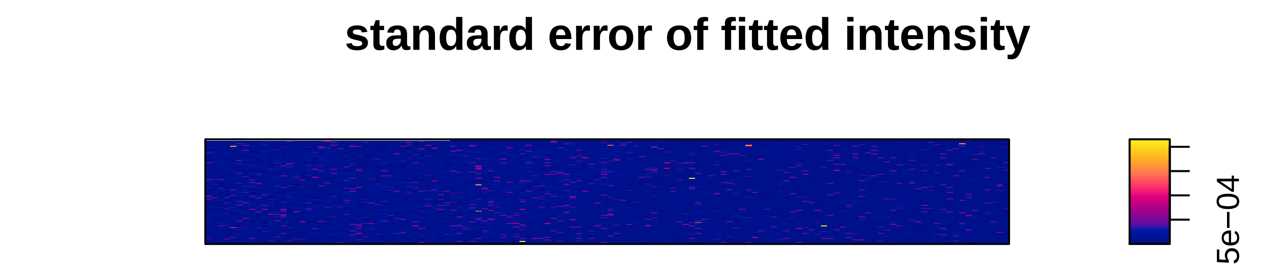
standard error of fitted intensity
5e−04

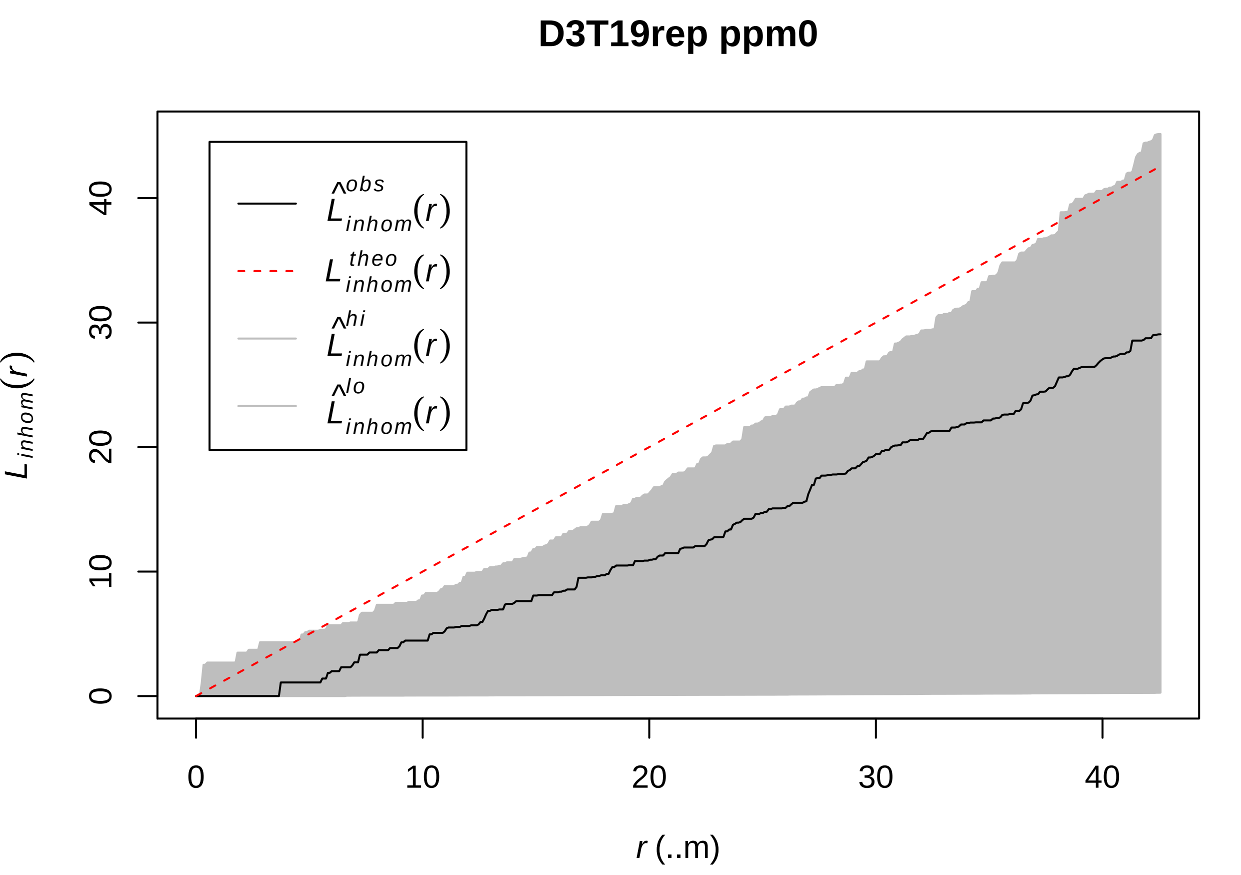
D3T19rep ppm0
$\hat{L}^{obs}_{inhom}(r)$
$L^{theo}_{inhom}(r)$
$\hat{L}^{hi}_{inhom}(r)$
$\hat{L}^{lo}_{inhom}(r)$
$L_{inhom}(r)$
0
10
20
30
40
r (..m)

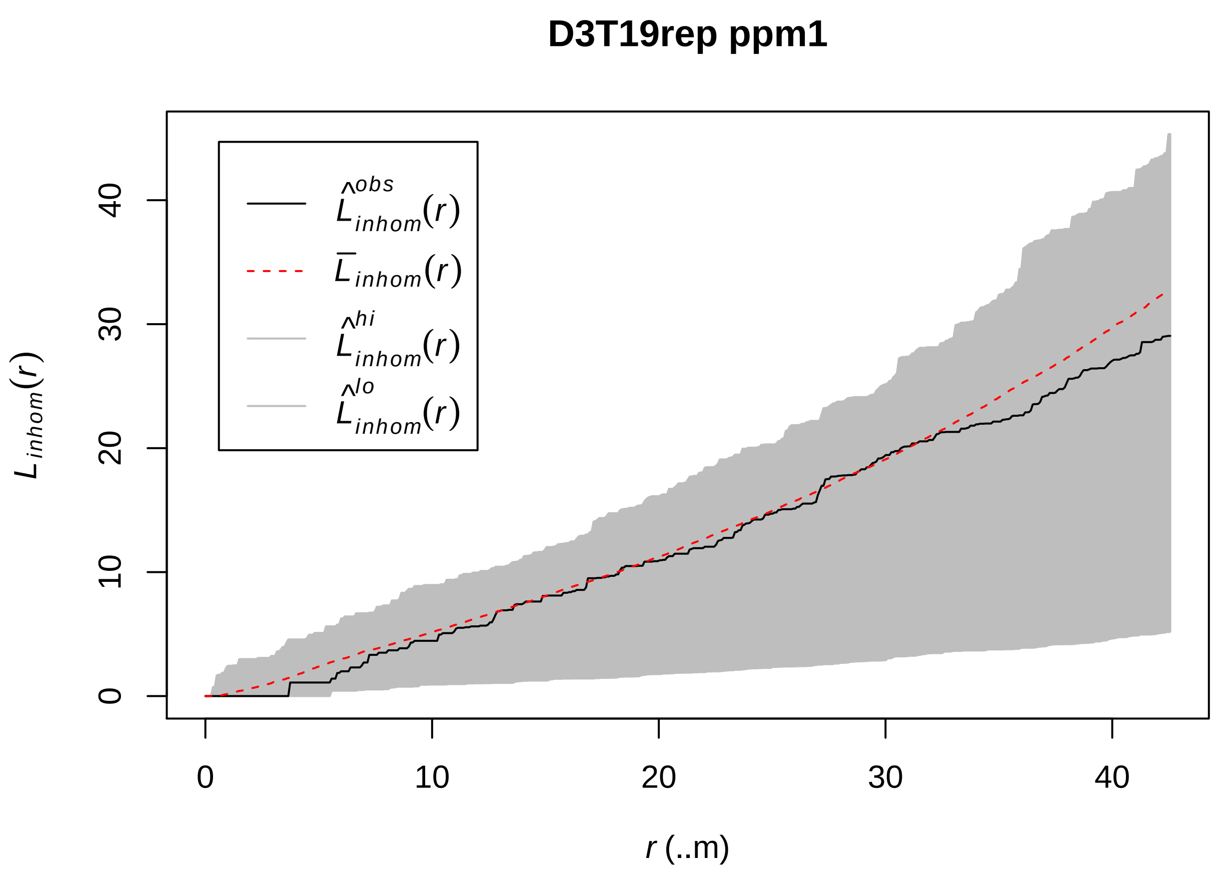
D3T19rep ppm1
$\hat{L}^{obs}_{inhom}(r)$
$\bar{L}_{inhom}(r)$
$\hat{L}^{hi}_{inhom}(r)$
$\hat{L}^{lo}_{inhom}(r)$
$L_{inhom}(r)$
0
10
20
30
40
r (..m)

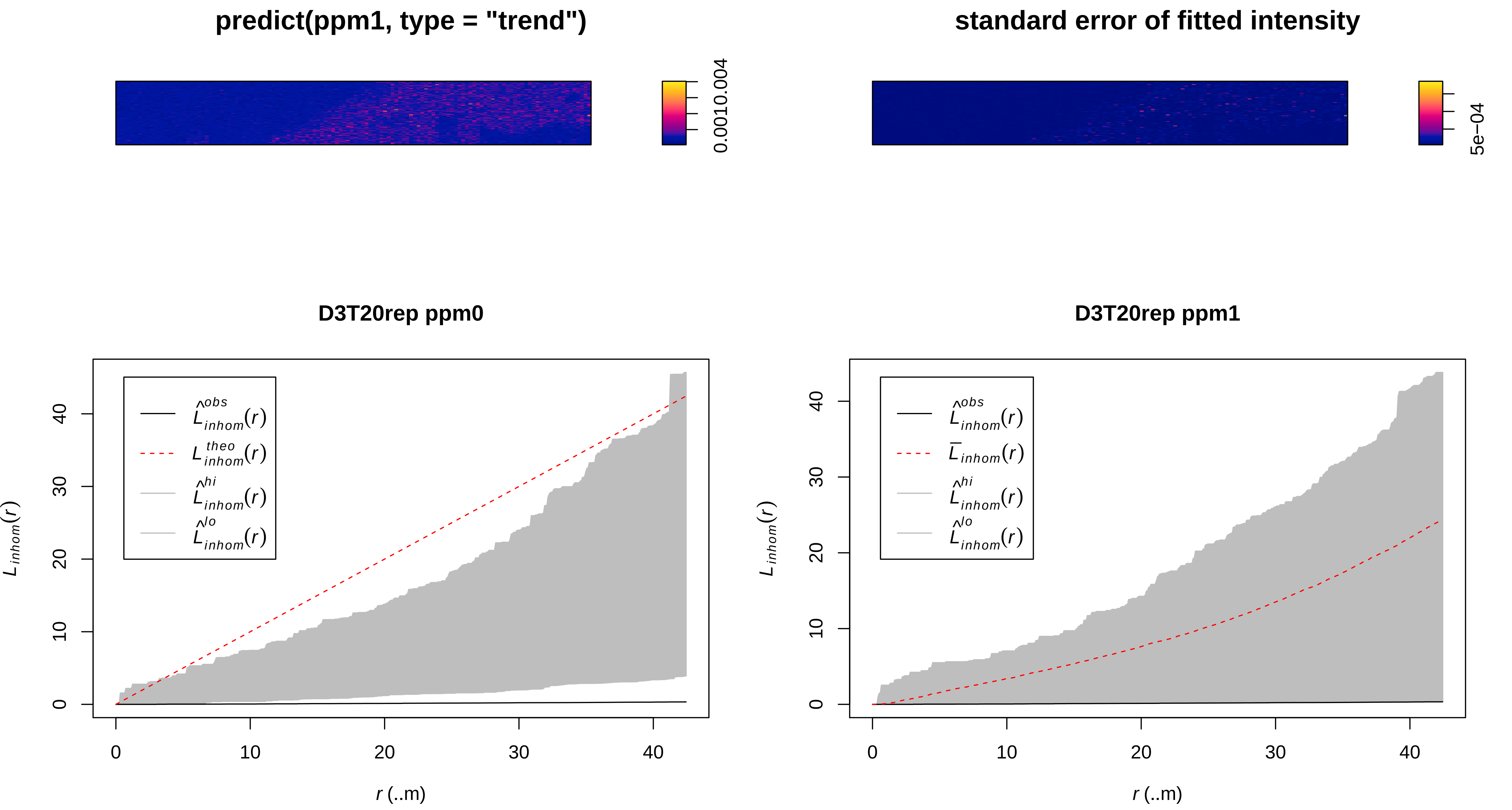
predict(ppm1, type = "trend")
0.001
0.004
standard error of fitted intensity
5e−04
D3T20rep ppm0
D3T20rep ppm1
$\hat{L}^{obs}_{inhom}(r)$
$L^{theo}_{inhom}(r)$
$\bar{L}_{inhom}(r)$
$\hat{L}^{hi}_{inhom}(r)$
$\hat{L}^{lo}_{inhom}(r)$
$L_{inhom}(r)$
r (..m)
0
10
20
30
40
