# Supplementary material for: Rock-Hosted Subsurface Biofilms: Mineral Selectivity Drives Hotspots for Intraterrestrial Life
Source: Front Microbiol. 2021 Apr 9;12:658988. doi: 10.3389/fmicb.2021.658988 (PMC8062869; doi:10.3389/fmicb.2021.658988)
Supplement: Supplementary file 3 [file Image_2.PDF]

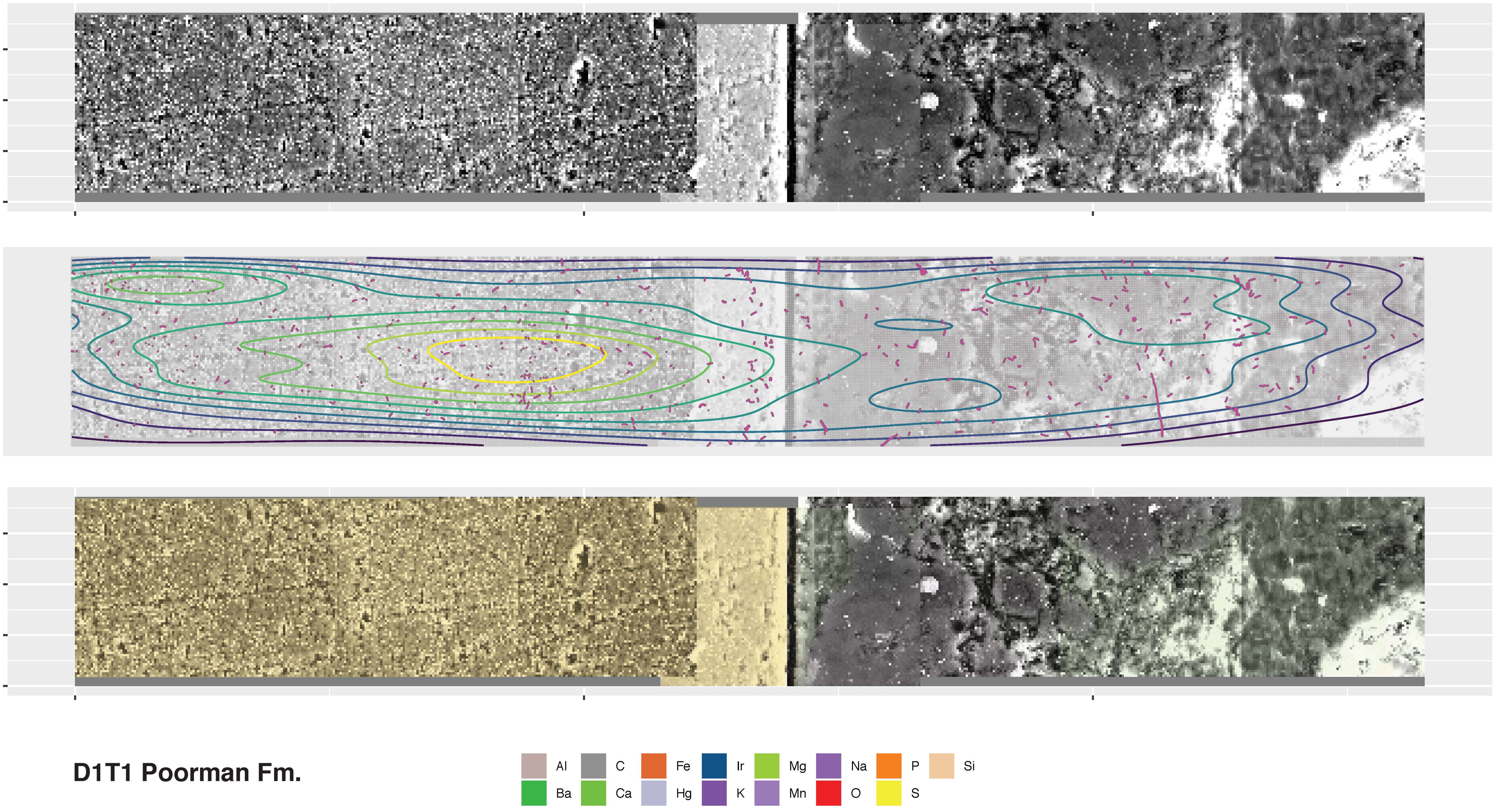
D1T1 Poorman Fm.
Al
C
Fe
Ir
Mg
Na
P
Si
Ba
Ca
Hg
K
Mn
O
S

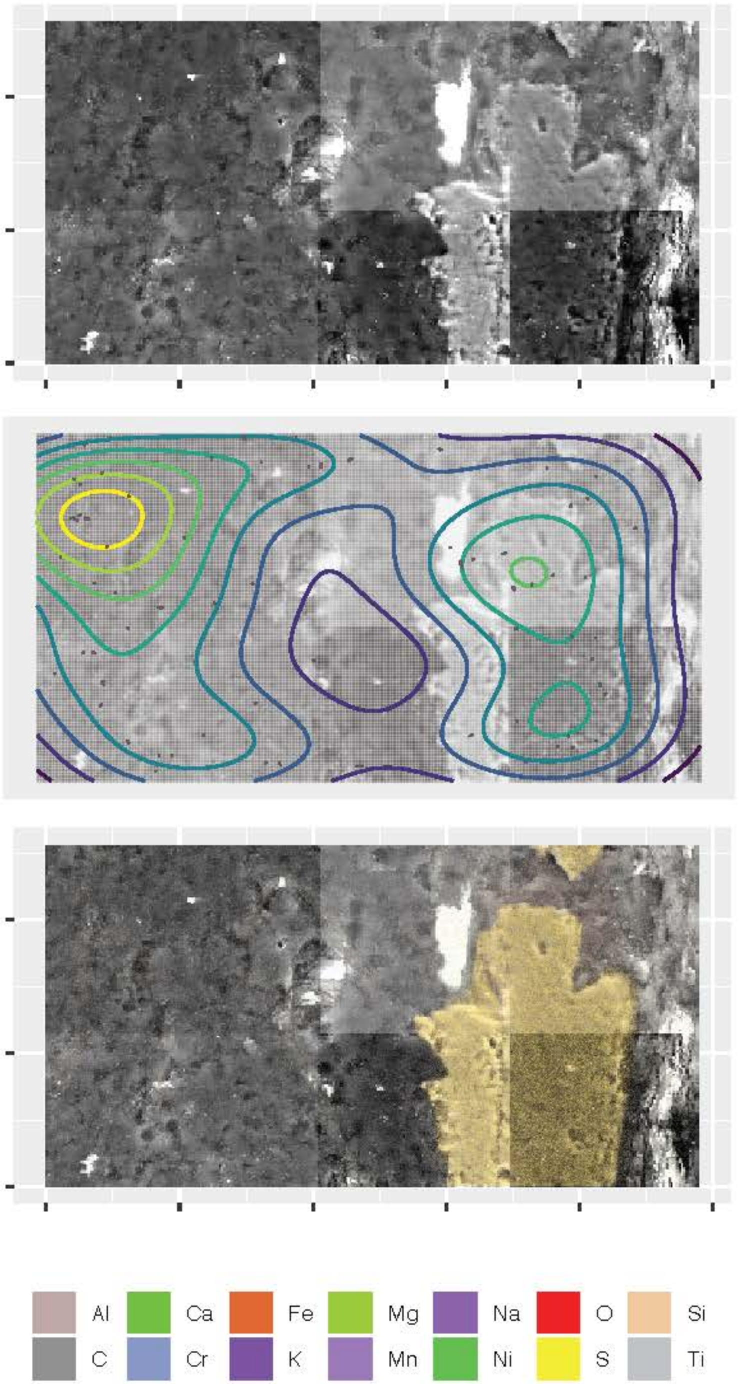

D1T2 Poorman Fm.

D1T3 Ellison Fm.

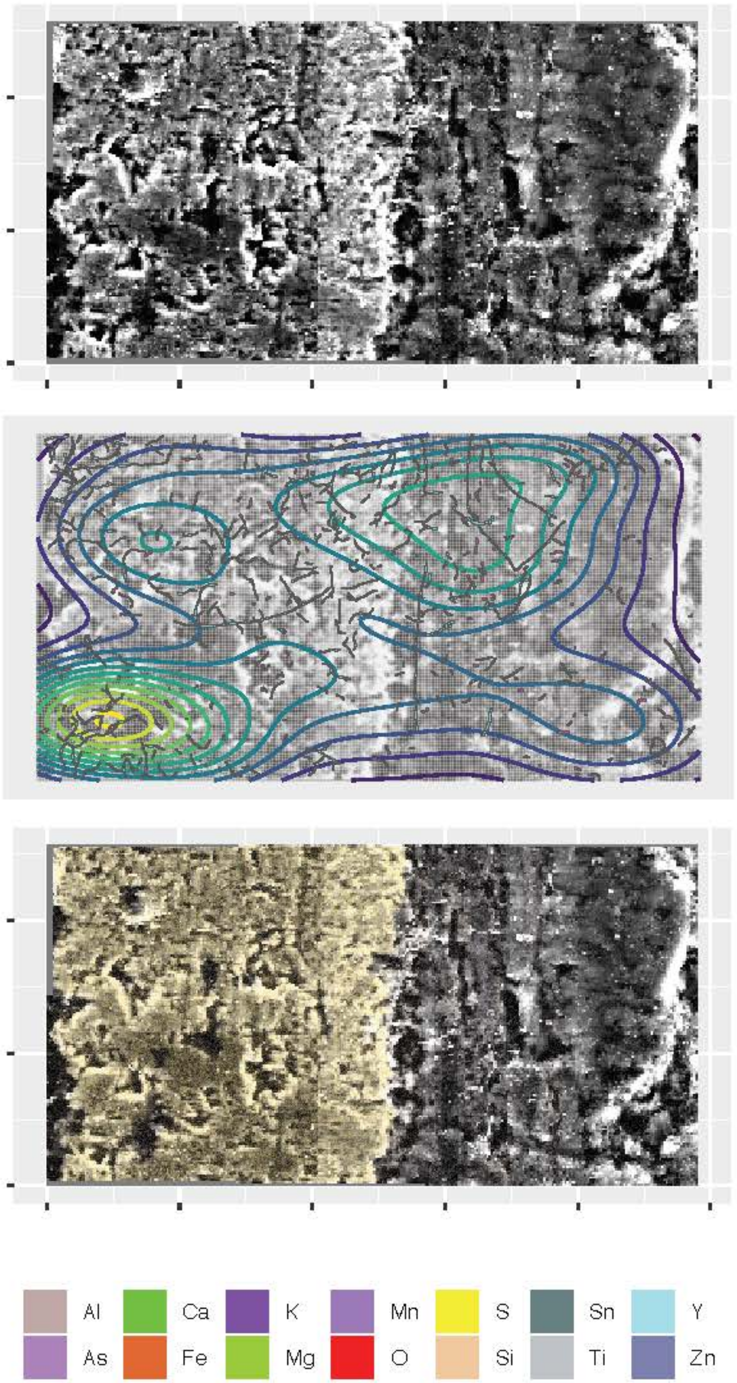

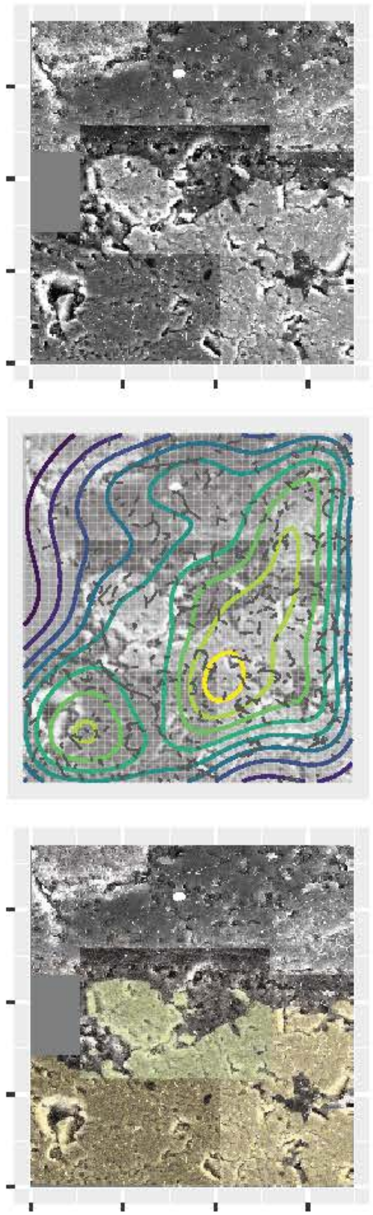

D1T4 Ellison Fm.

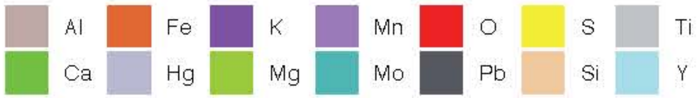

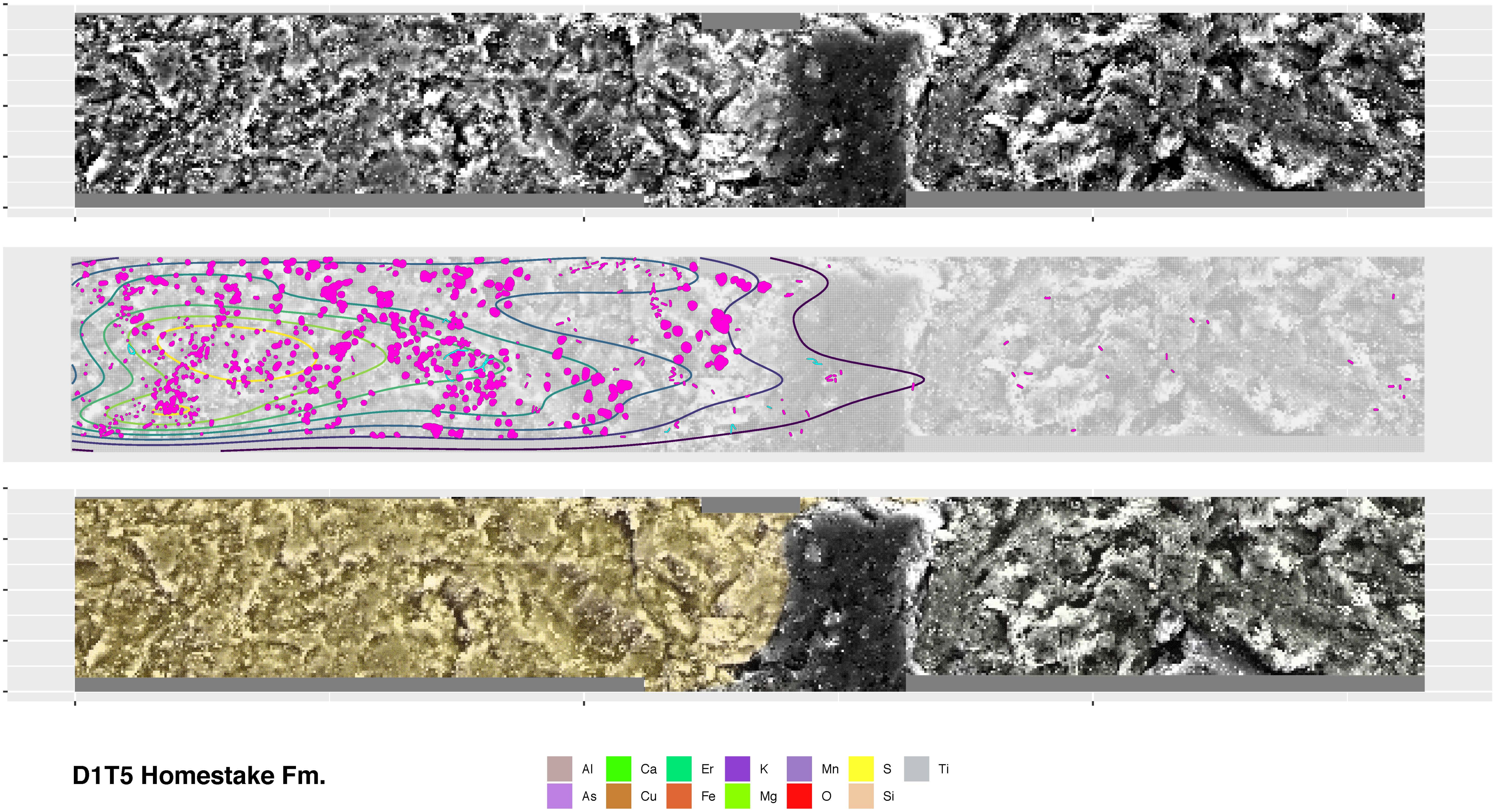

D1T5 Homestake Fm.
Al
As
Ca
Cu
Er
Fe
K
Mg
Mn
O
S
Si
Ti

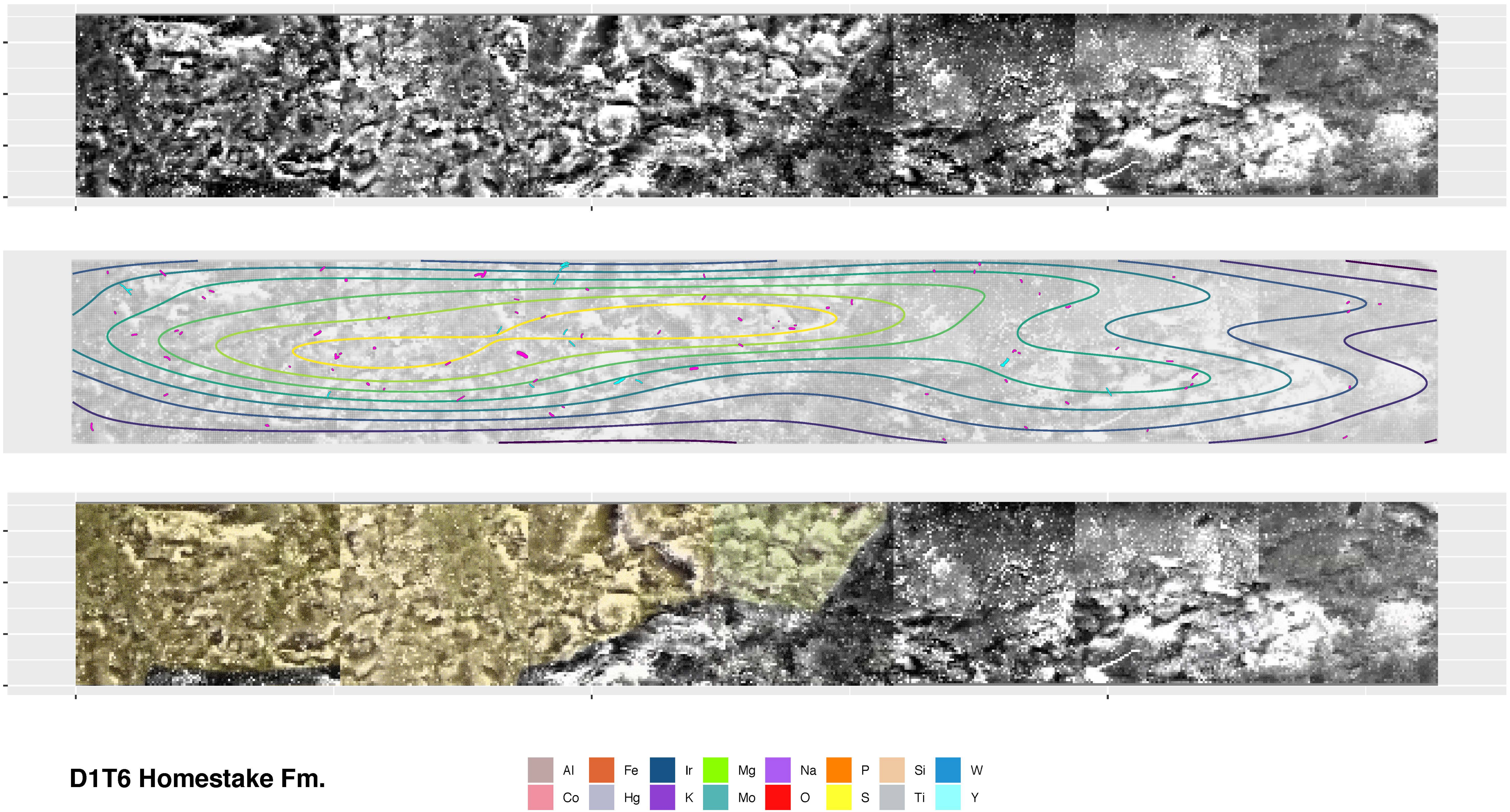
D1T6 Homestake Fm.
Al
Co
Fe
Hg
Ir
K
Mg
Mo
Na
O
P
S
Si
Ti
W
Y

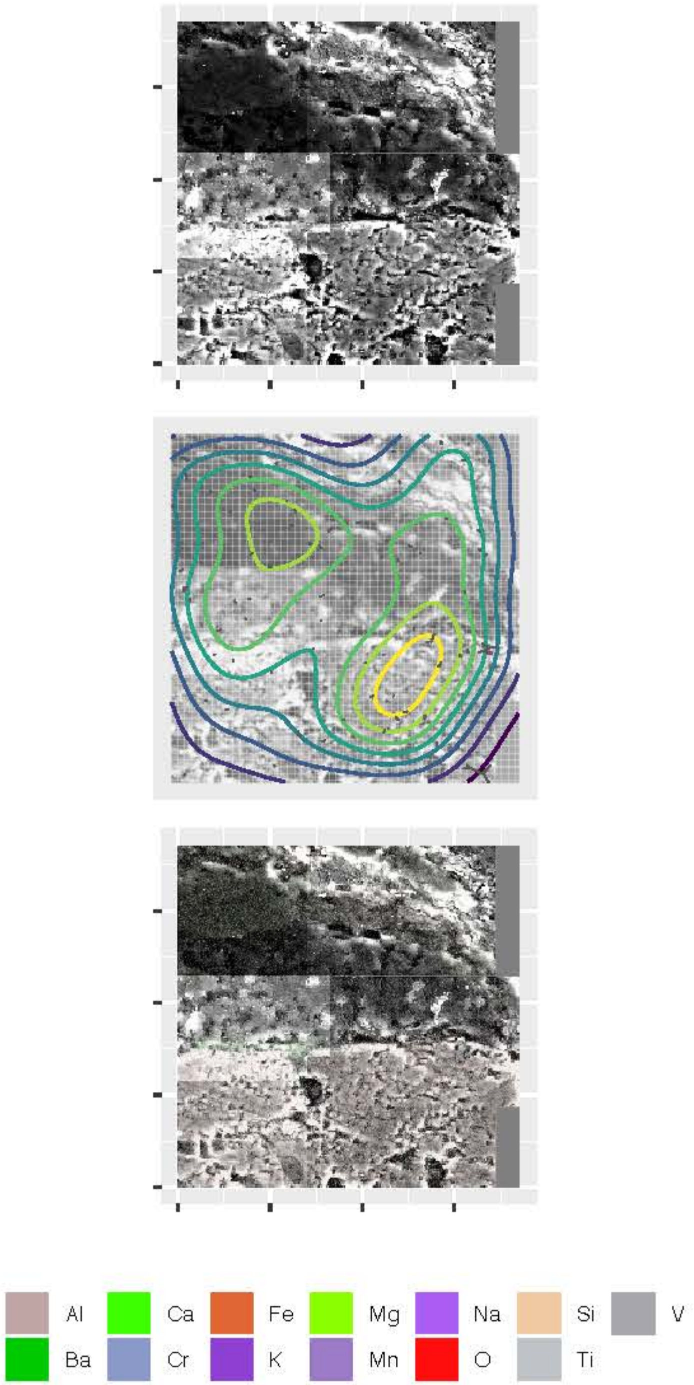

D1T7 Yates Unit

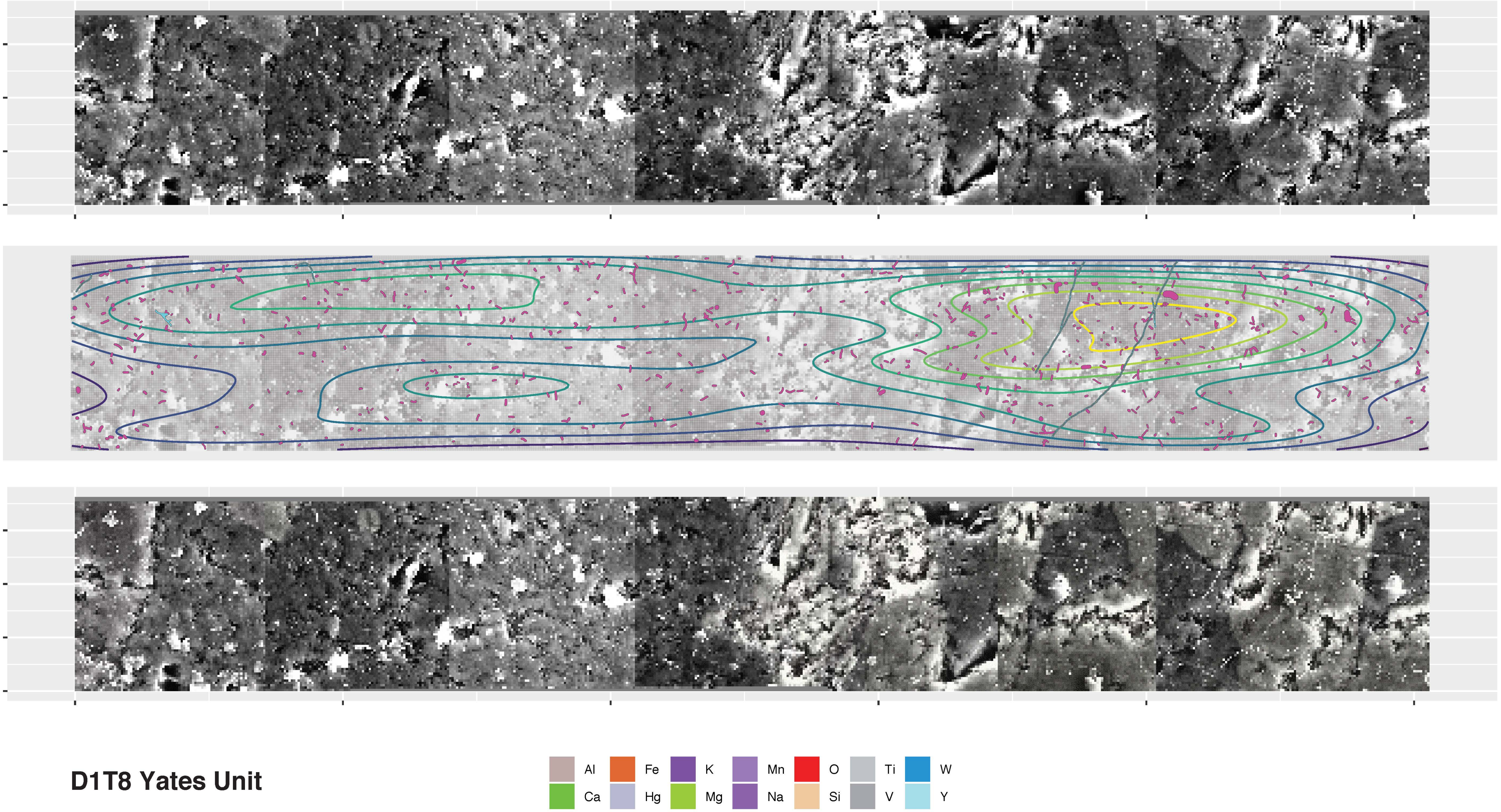

D1T8 Yates Unit

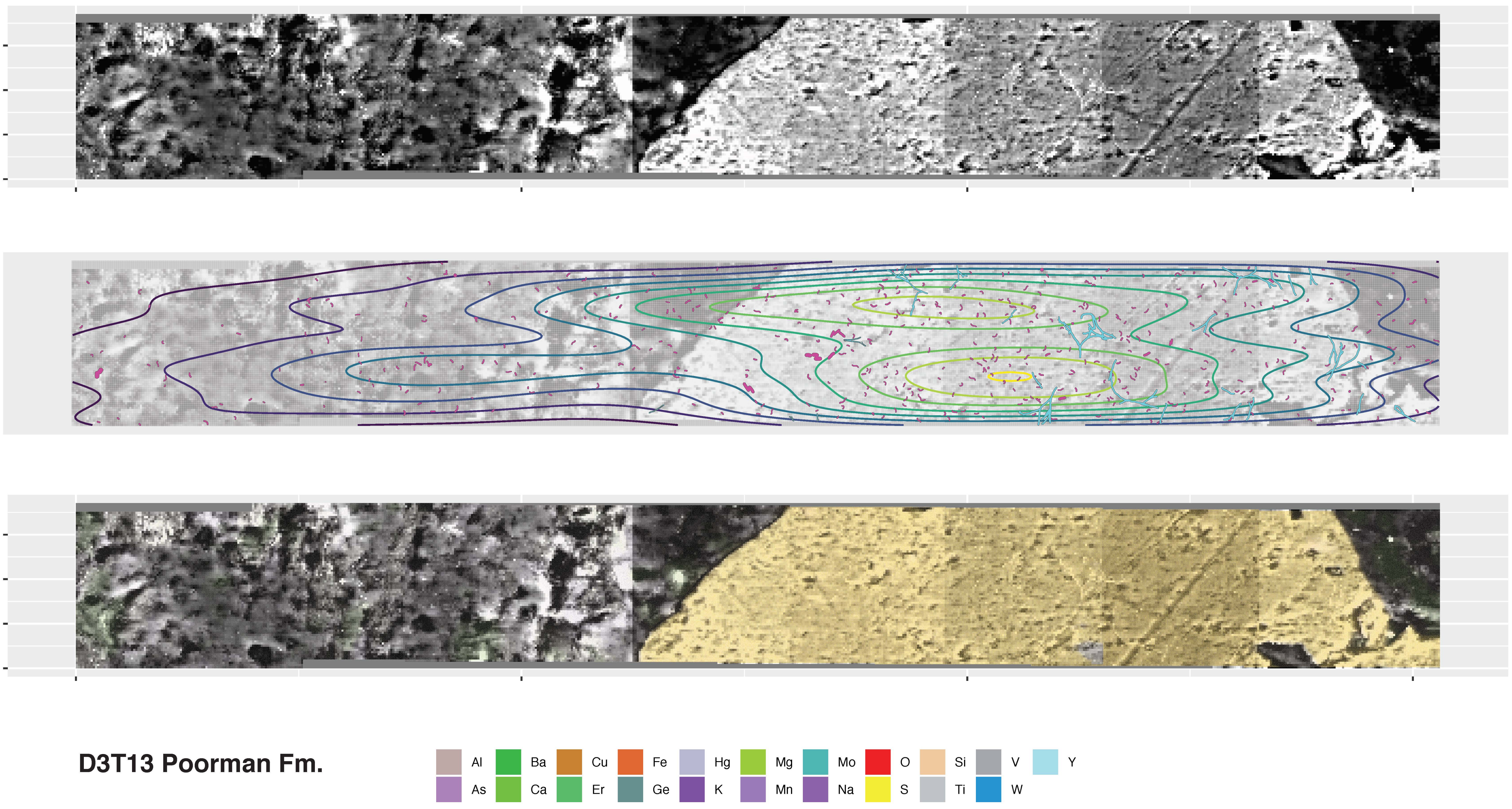

D3T13 Poorman Fm.
Al
As
Ba
Ca
Cu
Er
Fe
Ge
Hg
K
Mg
Mn
Mo
Na
O
S
Si
Ti
V
W
Y

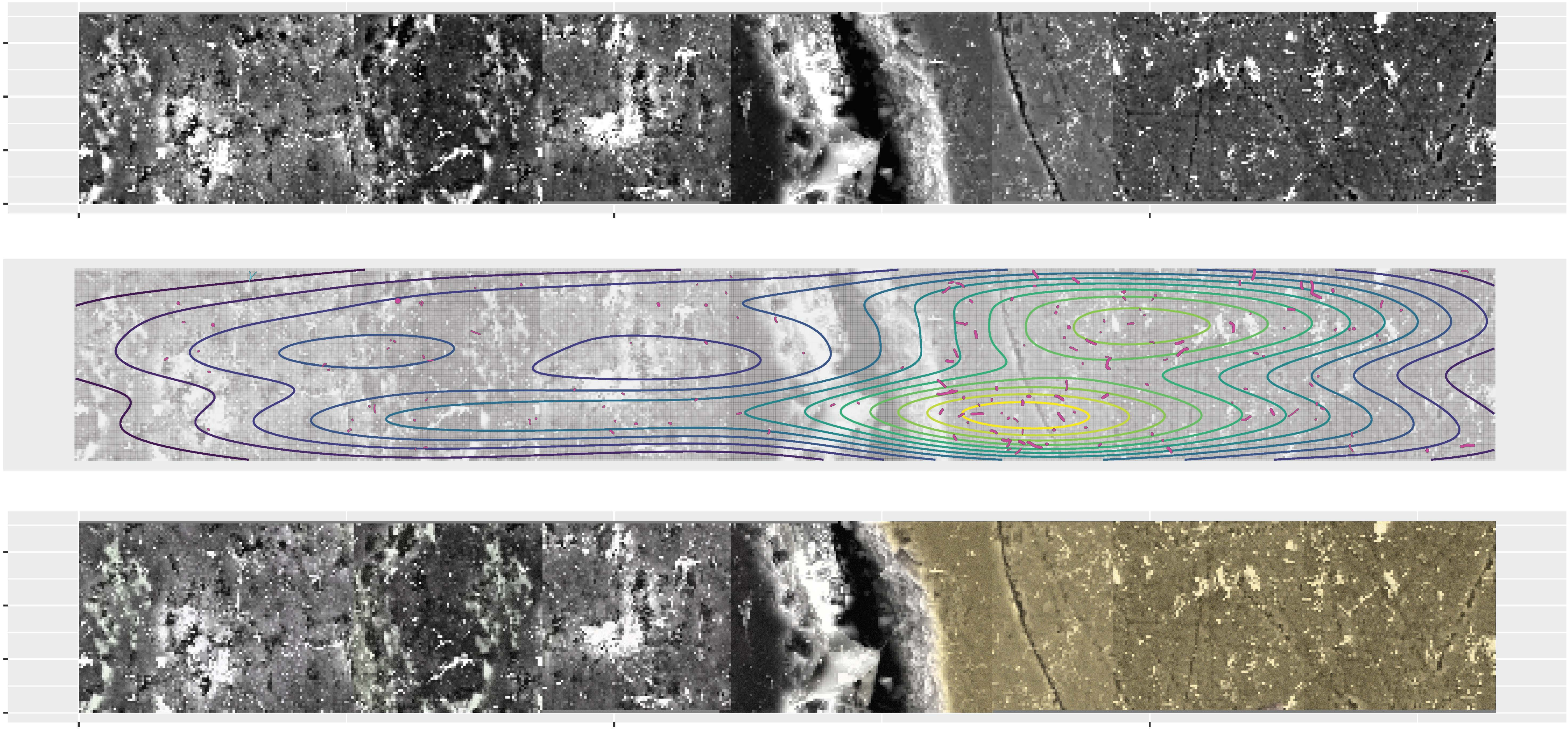

D3T14 Poorman Fm.

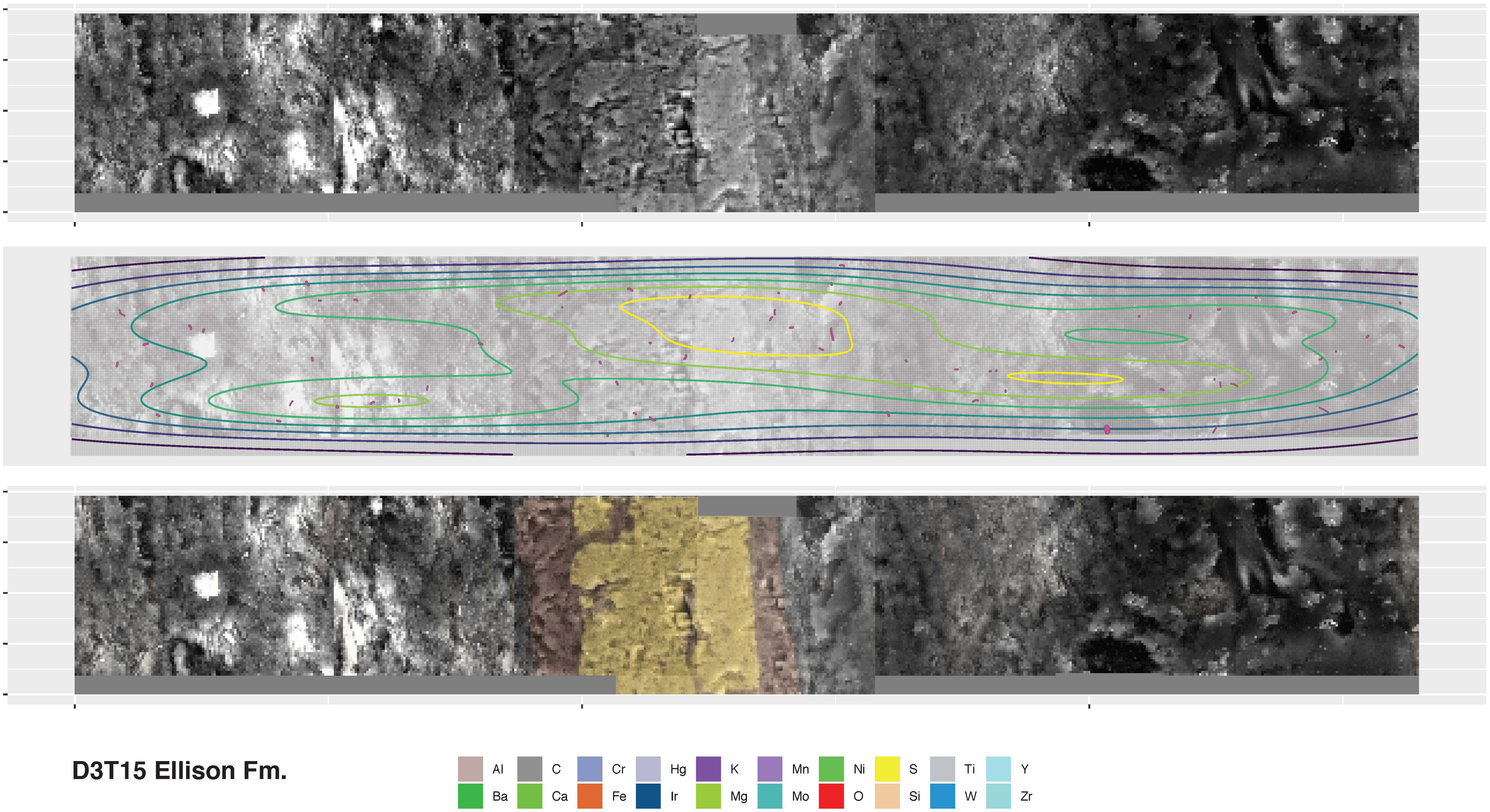

D3T15 Ellison Fm.

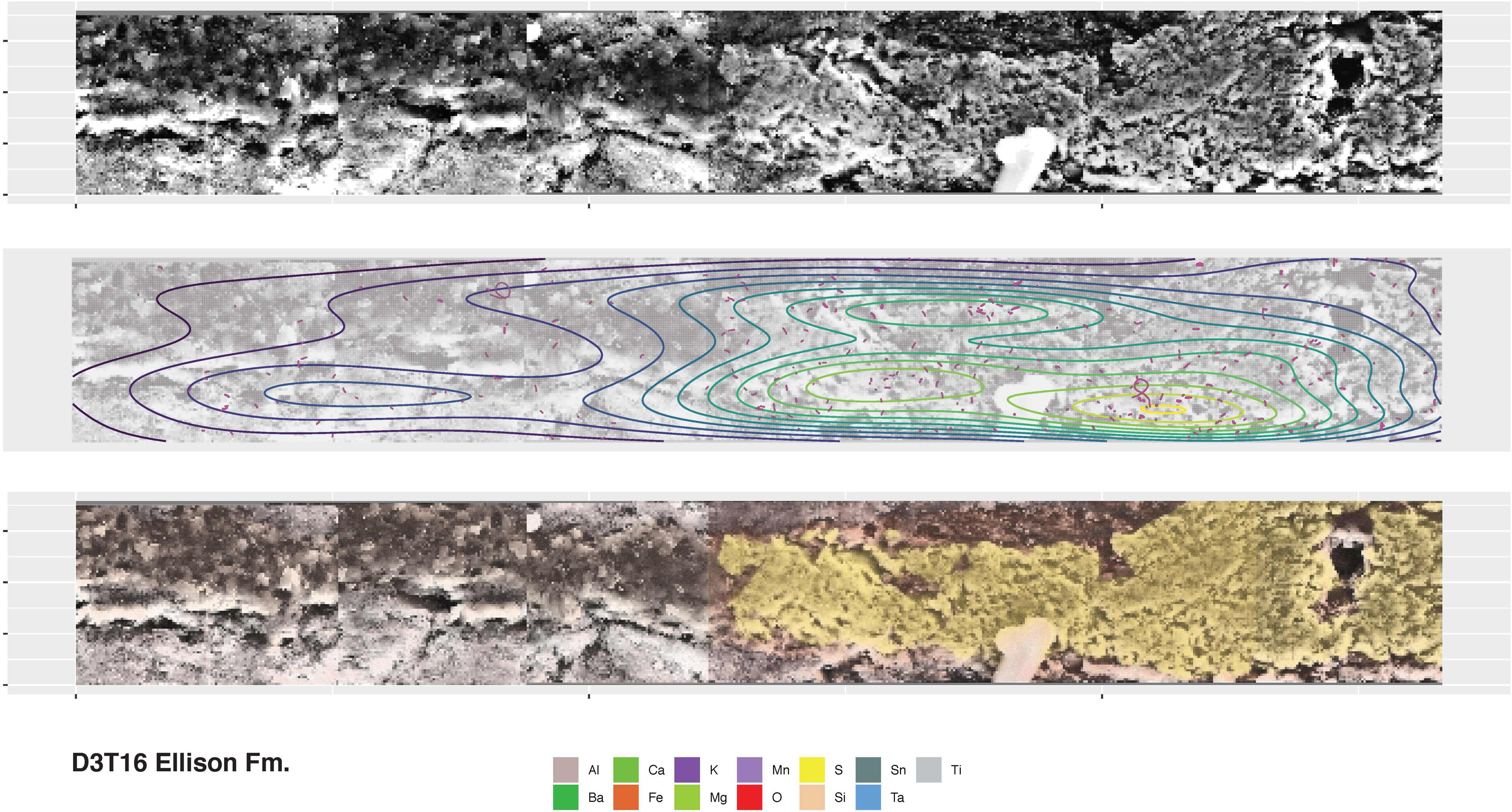

D3T16 Ellison Fm.

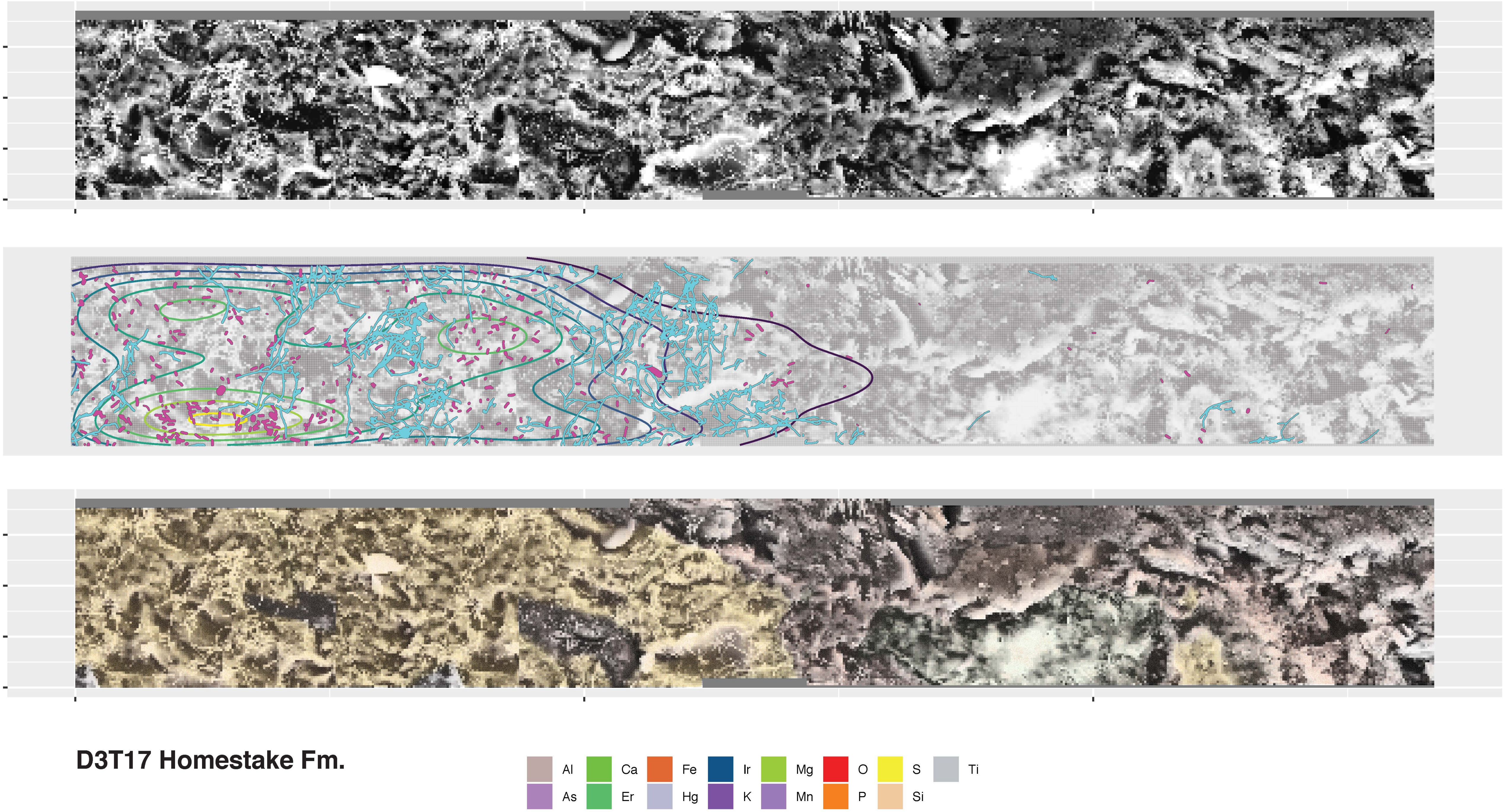

D3T17 Homestake Fm.

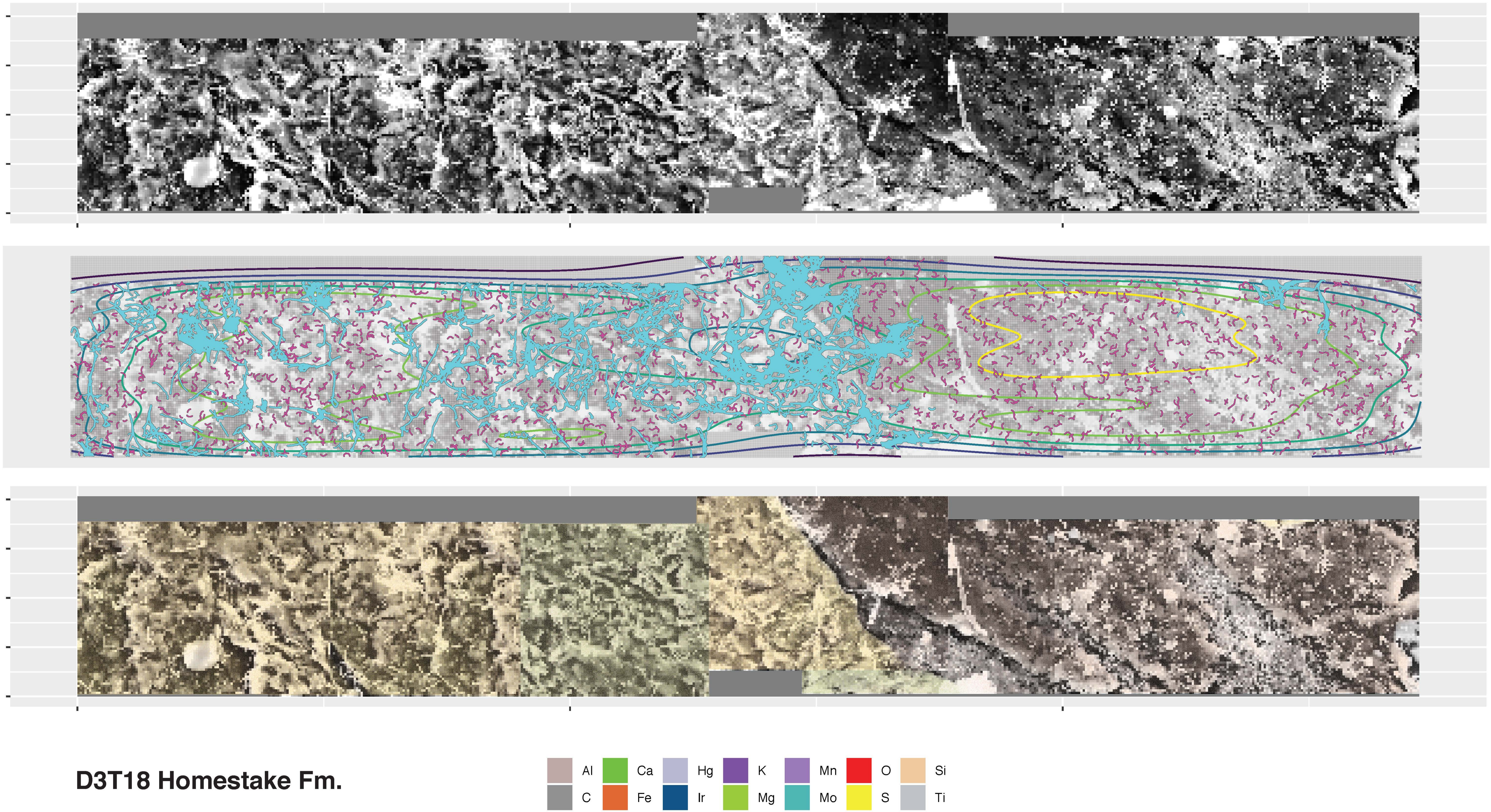
D3T18 Homestake Fm.
Al
C
Ca
Fe
Hg
Ir
K
Mg
Mn
Mo
O
S
Si
Ti

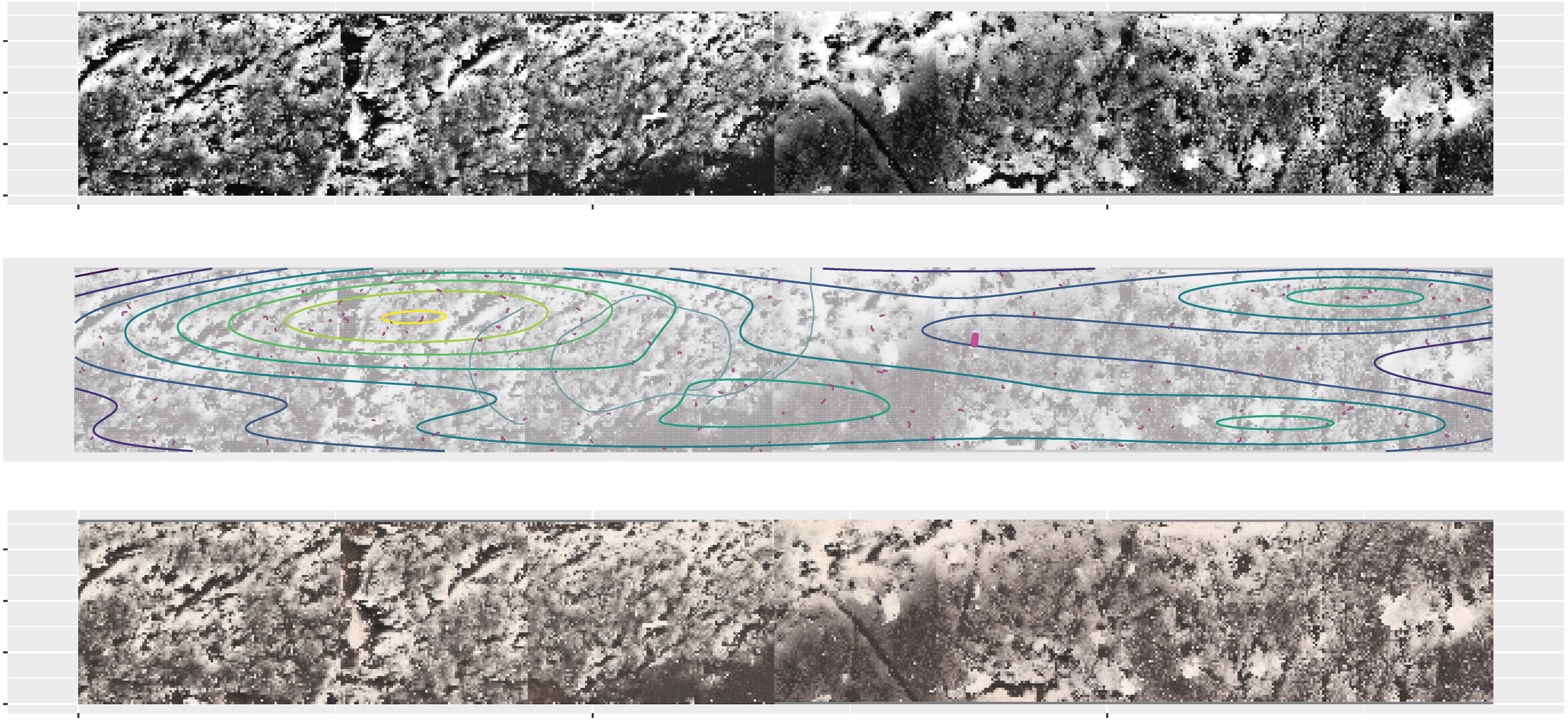

D3T19 Yates Unit

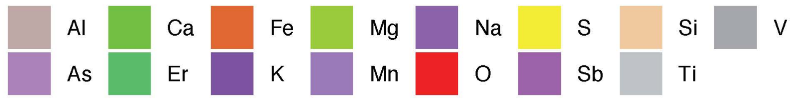

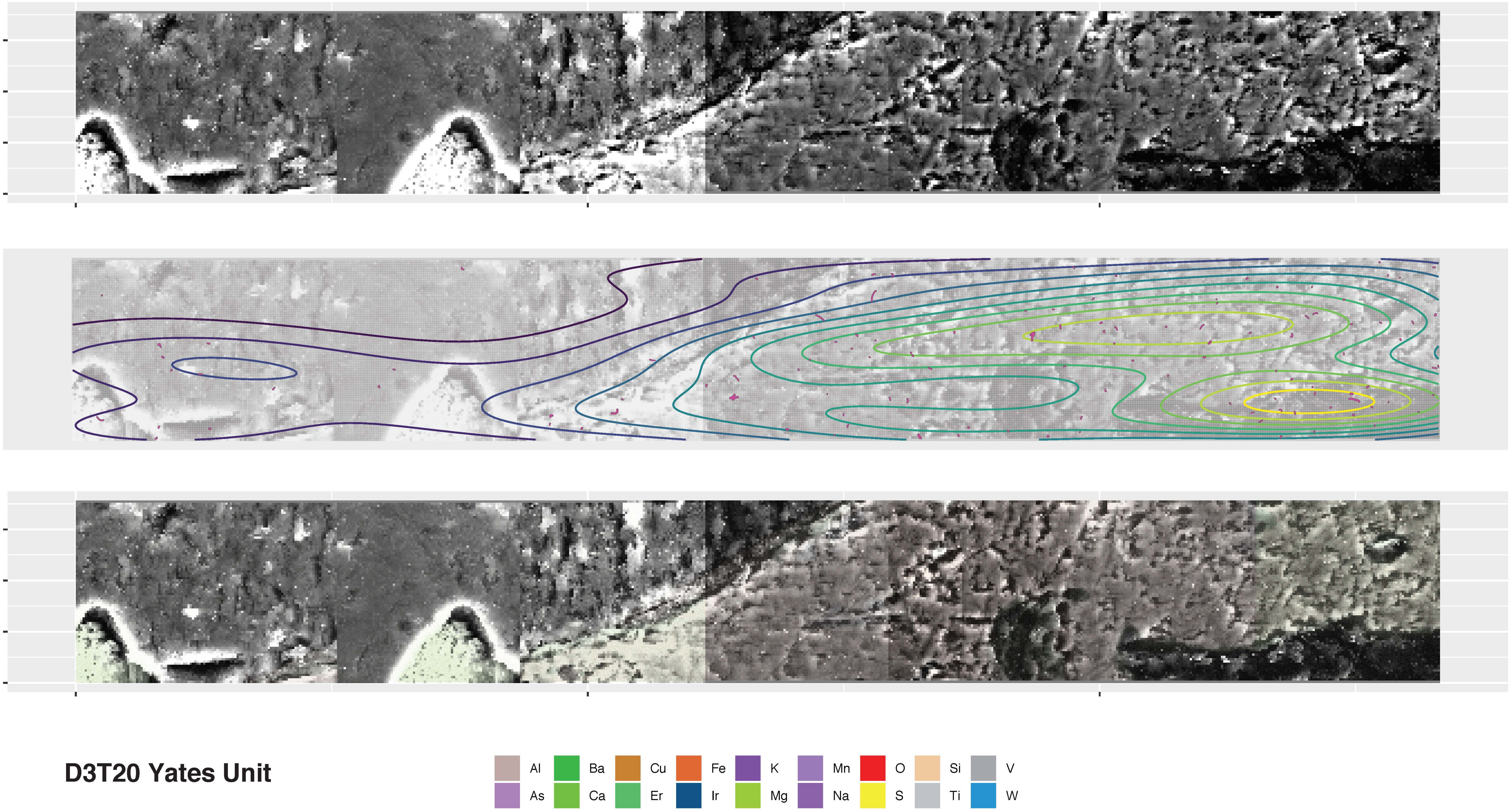

D3T20 Yates Unit
