# Supplementary material for: Rock-Hosted Subsurface Biofilms: Mineral Selectivity Drives Hotspots for Intraterrestrial Life
Source: Front Microbiol. 2021 Apr 9;12:658988. doi: 10.3389/fmicb.2021.658988 (PMC8062869; doi:10.3389/fmicb.2021.658988)
Supplement: Supplementary file 4 [file Image_3.PDF]

Supplementary Material

1 Supplementary Data

1.1 Supplementary Figures

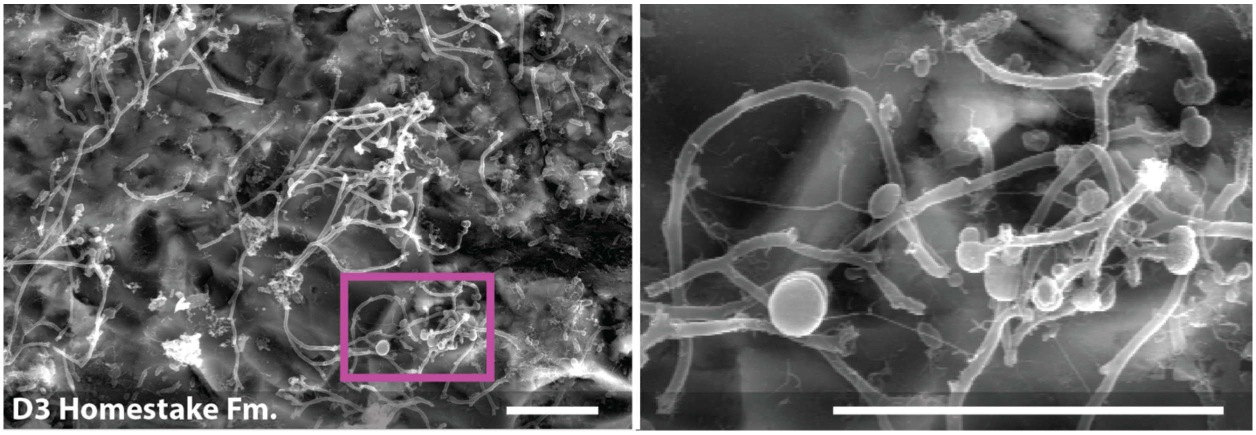

Supplementary Figure 1. Putative fungal hyphae colonizing Homestake Formation in D3 experiments. A region of putative fungal morphologies showing bifurcating filaments and round spore-like features (left). Scale bar represents 10 µm, purple box outlines the zoomed in area (right).

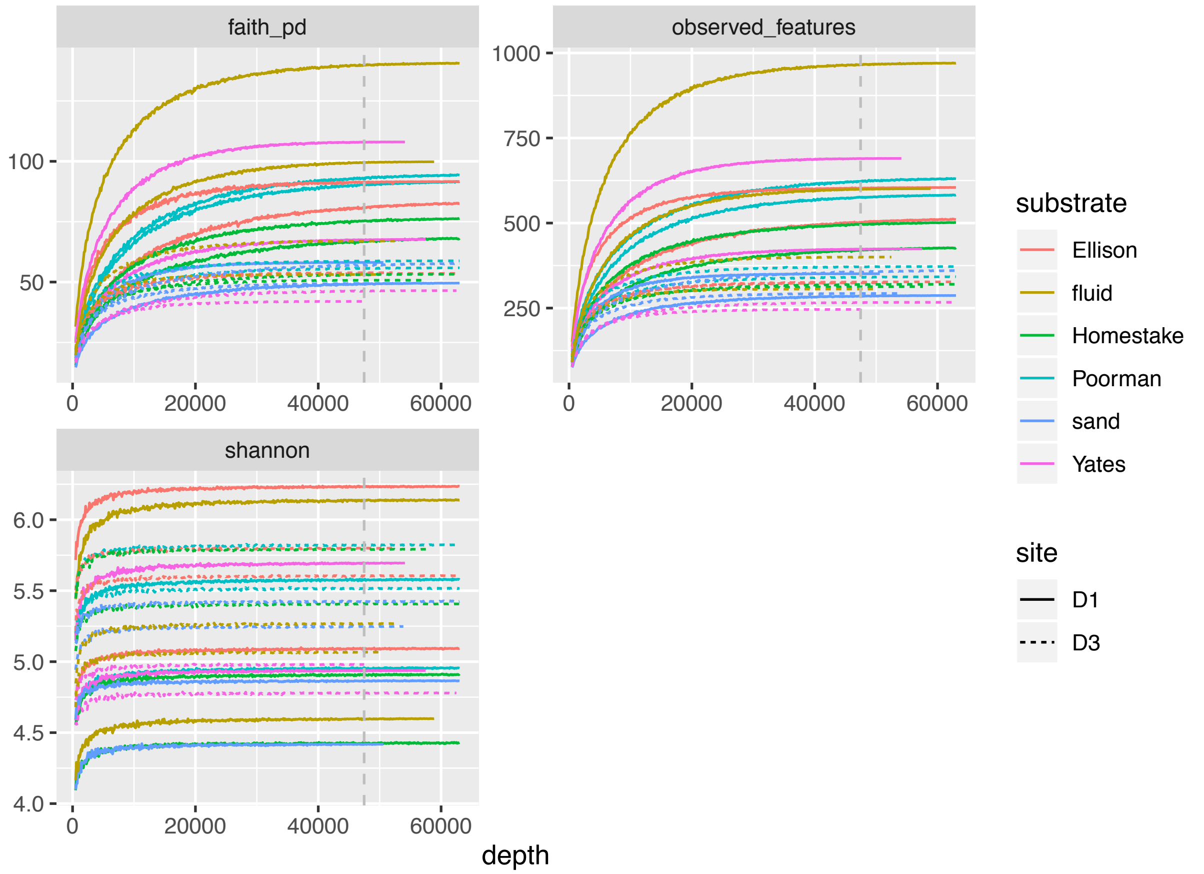

Supplementary Figure 2. Alpha diversity as rarefaction curves. Vertical dashed line denotes depth at which data were rarefied for this study.

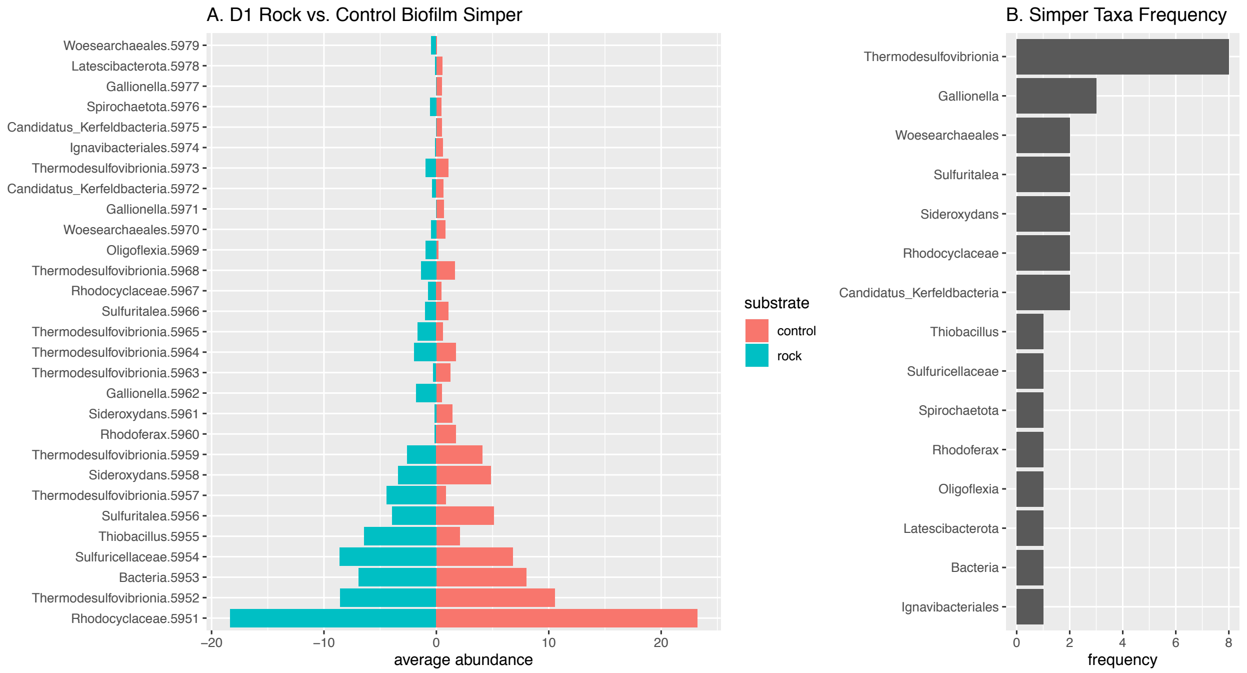

Supplementary Figure 3. A) SIMPER analysis of D1 rock vs. control biofilm communities. Only the taxa contributing to the top 75% of average between-group similarity are shown. Taxa are classified to the lowest possible level, numbers next to taxa names denote unique ASVs within the taxonomic group. B) Frequency of taxa shown in panel A.
